# DNA and RNA editing for the therapy of human diseases: current status, challenges, and future prospects

**DOI:** 10.1186/s43556-026-00456-x

**Published:** 2026-04-22

**Authors:** Rui Zhao, Changli Wang, Jiamei Li, Yan Liao, Chenwei Huang, Ting Hu, Haoling Zhang, Wangzheqi Zhang

**Affiliations:** 1 Gansu Province, Gansu University of Chinese Medicine, No.35 Dingxi East Road, Lanzhou, 730000 China; 2 Naval Medical University, No.800 Xiangyin Road, Shanghai, 200433 China; 3 Department of Biomedical Sciences, Cancer Research and Specialist Centre, Universiti Sains Malaysia, 13200 Penang, Malaysia

**Keywords:** DNA Editing, RNA Editing, Gene Therapy, CRISPR Technology, Precision Medicine

## Abstract

The rapid development of DNA- and RNA-editing tools (collectively referred to as gene editing technologies) has caused a paradigm shift in the treatment of human diseases from symptomatic treatment to precision-based medicine. Both DNA-based and RNA-based editing systems, including Clustered Regularly Interspaced Short Palindromic Repeats (CRISPR)–derived technologies and newly developed RNA editing tools, have pushed technological frontiers in terms of editing precision, hierarchical control, and reversibility; they have accumulated a growing body of preclinical and clinical evidence across diverse diseases ranging from inherited disorders to cancer, infectious diseases, and neurodegenerative diseases (ND). This review systematically summarizes the core principles and representative advances of DNA-based genome editing and RNA-based transcriptome editing technologies, comprehensively compares the two categories of technical strategies in terms of therapeutic potential, durability of effects, and risk profiles, and further explores the key challenges for achieving long-term safe and efficient in vivo applications, covering core bottlenecks such as delivery efficiency, tissue specificity, genotoxicity, and immunogenicity. Safety assessment has broadened to include tracking genotoxicity and genomic structural variations, whereas delivery systems and tissue specificity are determinant factors for in vivo therapeutic applications. Through the employment of both permanent and reversible editing strategies with high cargo-writing capacity and low integration risk, combined with programmable delivery systems, the therapeutic potential of hard-to-transfect tissues and complex diseases is anticipated to be broadened, opening new paths for clinical translation.

## Introduction

After the creation of programmable nuclease systems such as zinc finger nucleases (ZFNs) and transcription activator-like effector nucleases (TALENs), new genome editing technologies have continuously improved precision with reduced cytotoxicity, providing a basis for clinical application of DNA editing. In this review, we use the term “gene editing technologies” as a collective concept encompassing both DNA-level genome editing and RNA-level transcriptome editing. When referring specifically to permanent genomic modifications, we use “genome editing” or “DNA editing”, whereas “RNA editing” is used exclusively for post-transcriptional modifications without altering the genomic sequence. The emergence of the CRISPR system greatly increased editing efficiency, and following the development of base editors and prime editors, precise rewriting of DNA sequences without inducing DNA double-strand breaks (DSBs) became possible [1, 2]. Notably, PE uses a reverse transcription template–mediated programmable “search-and-replace” mechanism and can achieve single-base substitutions, insertions, and deletions within a single platform, thereby showing extensive potential applications in gene function studies and disease modeling [3, 4]. The parallel advancement of editing enzyme engineering and delivery systems has further promoted in vivo feasibility. Specifically, lipid nanoparticle (LNP)–formulated ribonucleoprotein (RNP) delivery, facilitated by ionizable lipid design and process optimization, is likely to increase editing efficiency while minimizing safety risks such as immune activation and random genomic integration [5, 6]. The clinical application of ex vivo CRISPR therapies in inherited hematologic disorders (such as the approval of Casgevy) has placed genome editing on a path toward comprehensive and systematic clinical evaluation; however, off-target effects, challenges in tissue-specific delivery, and long-term safety remain major obstacles to broader clinical translation [7]. In addition, emerging systems such as recombinases, mobile genetic elements, and epigenetic editors have extended gene manipulation beyond linear DNA sequences to chromatin architecture and transcriptional regulation, enabling more sophisticated layers of control [8–10].

Alongside DNA editing, RNA editing offers reversible and spatiotemporally controllable regulation of disease-relevant transcripts, which is particularly attractive in disorders requiring dynamic and fine-tuned intervention. Its scope has expanded substantially with engineered Cas13 systems, which have been applied to targeted RNA degradation and base modification in cellular and in vivo settings, while dCas13-based post-transcriptional platforms enable correction of aberrant splicing and modulation of transcript isoform ratios [11–13]. Notably, several compact Cas13 variants can be delivered in vivo using adeno-associated virus (AAV) vectors and efficiently silence target RNAs, highlighting the translational promise of RNA-targeting therapeutics [14, 15]. Together, DNA and RNA editing technologies are evolving as parallel and complementary modalities that establish a multilayered regulatory framework spanning the genome and transcriptome.

From the perspective of disease burden, genetic diseases, cancer, infectious diseases, and degenerative disorders remain major global health challenges, and current therapies are often limited by insufficient durability or mechanistic specificity. Monogenic diseases caused by well-defined pathogenic variants are particularly suitable targets for gene editing interventions. Increasing evidence shows that BE can correct disease-causing mutations without inducing harmful DNA DSBs while producing stable phenotypic improvement in animal models [16–18]. More broadly, both DNA- and RNA-based editing strategies offer distinctive advantages by intervening directly in pathogenic molecular processes, with demonstrated or emerging applications in inherited diseases, cancer immunotherapy, and infectious conditions [19–21]. These developments underscore the growing therapeutic importance of gene editing technologies across diverse disease settings.

Despite this rapid progress, the literature remains fragmented, with DNA and RNA editing technologies often discussed in isolation and translational challenges addressed in a modality-specific manner. A systematic and comparative synthesis that integrates mechanistic principles, therapeutic potential, and safety considerations across both DNA- and RNA-based editing platforms is therefore timely. Accordingly, this review aims to provide an integrated overview of recent advances in gene editing technologies, with particular emphasis on the complementary roles of DNA and RNA editing in disease therapy. This review introduces the core molecular architectures and engineering evolutions of DNA and RNA editing systems and provides a comparative analysis of their therapeutic characteristics, durability, and risk profiles. It further summarizes current preclinical and clinical applications across genetic diseases, cancer, infectious diseases, and neurodegenerative disorders, and examines key translational bottlenecks—including delivery efficiency, genomic and transcriptomic safety, immunogenicity, and regulatory considerations—together with emerging technological innovations and interdisciplinary strategies that may shape the future development of gene editing technologies within the framework of precision medicine. This synthesis delineates the molecular architecture and engineering evolution of DNA and RNA editing toolkits, mapping their transformative impact across a diverse disease spectrum—from monogenic and oncological pathologies to infectious and neurodegenerative states. Beyond cataloging advances, the discourse interrogates the fundamental bottlenecks restricting clinical translation, framing the strategic imperatives required to bridge bench-side innovation with bedside reality. By anchoring the discussion within a "mechanism-application-barrier" nexus, this review forges an integrated paradigm that aligns technological prowess with the rigorous demands of genomic medicine.

## Overview of DNA and RNA editing technologies

Gene editing technologies have rapidly evolved into a diverse toolkit for precise intervention at both the genomic and transcriptomic levels. According to the molecular substrate and mode of action, current platforms can be broadly classified into DNA editing and RNA editing. DNA editing aims to achieve durable or permanent correction of pathogenic variants through direct modification of genomic sequences, whereas RNA editing enables reversible and programmable regulation at the transcript level. Recent advances in base editing, prime editing, endogenous enzyme recruitment, CRISPR-associated effectors, and delivery optimization have greatly expanded the scope and translational potential of both strategies. Therefore, a systematic overview of DNA and RNA editing technologies is essential for understanding their technical progress, biological features, and therapeutic prospects.

### Recent advances in DNA editing tools

DNA editing is a technological framework for permanent genomic modification and represents one of the core strategies for correcting pathogenic mutations at their source. Within the broader landscape of DNA and RNA editing, DNA editing enables direct rewriting of genomic sequences to achieve durable correction of disease-causing variants. Current development priorities therefore emphasize improving editing precision, reducing risks associated with DSBs, and enabling technically feasible in vivo applications. In recent years, the emergence of base editing (BE), prime editing (PE), and new site-specific sequence integration strategies has expanded DNA editing beyond conventional cut-and-paste paradigms, enabling single-nucleotide substitution, small-sequence rewriting, and targeted insertion of larger DNA cargos. At the same time, advances in Cas9 variant engineering and delivery optimization have broadened the range of targetable loci and tissues, thereby providing a foundation for systematic evaluation of editing efficiency across disease models [22, 23].

PE enables reverse transcription–guided sequence writing and can mediate multiple forms of DNA editing, including base substitutions and small insertions/deletions, without inducing DSBs[2]. This has substantially improved editing accuracy and expanded the spectrum of targetable mutations, highlighting its therapeutic potential for hundreds to thousands of pathogenic variants [2]. To standardize BE application, studies have systematically summarized the design and workflow of adenine base editors and cytosine base editors in mammalian cells, identifying key determinants of target selection, editor choice, and delivery strategy [24]. Current cytosine base editors (CBEs) and Adenine Base Editor (ABE) platforms can mediate efficient single-base substitutions in vitro and, in selected in vivo models, support therapeutically relevant editing with reduced DSB-associated risks, supporting their development as therapeutic tools for monogenic diseases [24, 25].

At the level of disease model validation, ABE8e-Nuclear localization–regulated Cas9-HF (NRCH) has been used to precisely convert the pathogenic Hepatitis B virus (HBV) binding site (HBBS) allele into the nonpathogenic Makassar variant Hb G-Makassar (HBBG) in hematopoietic stem/progenitor cells (HSPCs) derived from patients and in humanized sickle cell disease (SCD) mice. Following long-term transplantation, stable expression was maintained, markedly alleviating erythrocyte sickling and related pathological features without Cas9 nuclease-induced p53 activation or large deletions, supporting BE as a potentially curative one-time intervention for SCD [26]. In lipid metabolism and cardiovascular research, LNP-mediated delivery of CRISPR base editors achieved efficient liver editing of Proprotein Convertase Subtilisin/Kexin Type 9 (PCSK9) in cynomolgus monkeys, resulting in sustained reductions in plasma PCSK9 and Low-Density Lipoprotein (LDL) cholesterol after a single injection, thereby providing key evidence for single-intervention control of cardiovascular risk factors and the clinical feasibility of in vivo BE [27]. In central nervous system (CNS) work, a blood–brain barrier–penetrant AeCBE system achieved brain-wide C-to-T editing within Myocyte Enhancer Factor 2 C (Mef2c) mutant mice and corrected autism spectrum disorder behaviours, providing evidence of the ability of BE to enable in vivo correction of single-nucleotide mutations in the nervous system [28]. In X-linked adrenoleukodystrophy (ALD) models, patient-derived fibroblasts and mice were edited via co-administration of BE and homology-independent targeted integration (HITI) using AAV9 vectors. This approach restored ATP-binding cassette sub-family D member 1 (ABCD1) expression and rescued very long-chain fatty acid catabolism, suggesting a paradigm for mutation-specific RNA Editing for Specific C-to-U Exchange (RESCUE)-based development against monogenic diseases [29].

And with these, troubles in the accessibility to target and precision of editing are confronted thanks to constant progress in Cas9 variants or new BE systems. Cytosine and adenine base editors based on the compact Streptococcus sinensis SsiCas9 scaffold recognize an NNAAAA protospacer adjacent motif (PAM), while maintaining low off-target activity and low indel frequencies in human cells and early mouse embryos, thus significantly expanding the targetable sequence space in PAM-constrained systems [30, 31]. Versatile fusion of optimized Engineered Escherichia coli tRNA adenosine deaminase variant 8e (TadA8e) derivatives with near–PAM-free SpRYn allows highly accurate A-to-G, C-to-T substitutions and dual-base editing within a narrow editing window in plants; such highly precise tools exhibit relaxed PAM requirements for fine-tuning protein function as well as gradual trait improvement based on point mutations [32]. At a broader genomic level, the engineered SpCas9 variants SpG and near-PAM-free SpRY can greatly relax the PAM dependence, enabling nucleases and base editors to target almost genomewide loci in human cells, thus broadening the range of accurate DNA editors at disease-relevant targets [33].

In addition, the development of PE systems for molecular configuration and the provision of methods has been continuously improved. An NLS-enhanced SpCas9-mediated prime editor along with a split-intein adaptor allows adult mouse–editing to both correct pathogenic alleles and model tumors by dual-AAV delivery, which enhances the efficiency of editing, and facilitates in vivo PE [34]. Based on these reports, a process has been proposed—similar to the incorporation of 3′-truncated gRNA—in which structured RNA motifs are introduced at the 3′ end of pegRNA for epegRNA production. This approach enables stable PE induction that is three- to fourfold more efficient than unmodified pegRNAs in human cells without defined off-target effects, having successfully corrected various pathogenic mutations [35]. Through CRISPRi-based screening, the mismatch repair (MMR) pathway has been identified as a limiting factor of PE activities; therefore, the PE 4 and PE max systems dramatically enhance efficiencies for substitution and small indel sequences in a broad range of mammalian cells while maintaining precise editing and avoiding indels [36]. A split prime editor was able to precisely target endogenous Dnmt1 in human cells and adult mouse retina by dual-AAV delivery when using the Rma intein, presenting a potential path for application in adult tissues [37]. Furthermore, a streamlined split system (CC-PE) constructed with coiled-coil hetero-dimerization achieves editing efficiencies that are as good or better than those obtained by full-length prime editors in various cell types and mouse liver and can be equipped with either SpCas9-NG or SpRY to broaden the editable scope, gaining an advantage of compatibility with delivery methods and in vivo flexibilities [38].

In combination, the DNA editing toolbox now covers a broad spectrum of editing capabilities and has validated the approach (feasibility) for functional correction across various disease models. Together with a lower dependence on repetitive DSBs, loosening PAM requirements and development of split delivery and new integration strategies, the DNA editing field is improving in precision, target choice and adaptability in vivo, step by step reaching the technical specifications required for genome analysis improvement and reduction of complexity in complex genetic backgrounds and long-term studies. However, the collection of structural variants, uniformity of delivery and lifelong safety are still major problems that will directly impact clinical translation in terms of where it can and cannot go.

### Innovative development of RNA editing tools

Unlike the permanent intervention of DNA editing, RNA editing provides a reversible transcript-level regulatory strategy, offering unique advantages in disease settings that require high safety and temporal control. Because it does not alter genomic sequences and allows reversibility and spatiotemporal regulation, RNA editing represents a flexible approach for modulating disease-relevant genes. In recent years, major progress has been achieved in RNA base substitution, splicing modulation, and RNA-directed silencing, collectively expanding the RNA-editing toolbox in terms of diversity, specificity, and in vivo applicability [13, 39–41].

A single programmable RNA editing framework can enable both A-to-I and C-to-U RNA base conversion. The RESCUE platform, developed through directed evolution of Adenosine Deaminase Acting on RNA (ADAR) 2, provided a key proof of concept for this strategy, and subsequent studies have advanced dual-capable RNA base editing toward in vivo application. Collectively, these advances expand the range of targetable mutations and broaden the landscape of RNA function regulation [39, 42, 43]. Building on this concept, the Leveraging Endogenous ADAR for Programmable Editing of RNA (LEAPER) 2.0 system, which employs covalently closed circular ADAR-recruiting RNAs (circ-arRNAs), showed markedly improved A-to-I editing efficiency and reduced bystander editing in vitro and in vivo. In a humanized Hurler syndrome mouse model, AAV-delivered LEAPER 2.0 corrected a pathogenic point mutation, restored α-L-iduronidase activity, and reduced glycosaminoglycan accumulation, thereby demonstrating the therapeutic potential of endogenous ADAR targeting [44, 45]. The Assisted Model Building with Energy Refinement (AMBER) editor, further optimized to gain C-to-U catalytic activity, can accurately repair pathogenic transcripts with 8% to 38% efficiency in various cellular backgrounds and in mice, and meanwhile underscores a low level of off-target activity. This provides a potential RNA therapeutic approach to correcting T-to-C mutations [46]. Based on AI-assisted design, a platform termed ProAPOBECs was developed by fusing Apolipoprotein B mRNA Editing Catalytic Polypeptide-like (APOBEC) variants with PUF proteins. This platform enables efficient in vivo C-to-U editing of PCSK9 and Mef2c transcripts in mice, resulting in the amelioration of hypercholesterolemia and autism-like phenotypes. These findings demonstrate the therapeutic applications of designed RNA base editors for intervention in genetic diseases [42]. For further applications requiring editing specificity, the CLUSTER system introduces G·U wobble base pairing in guide RNAs and is artificially locked in a circular conformation that efficiently recruits endogenous ADAR to achieve highly site-specific A-to-I editing of the pathological mutations within Methyl-CpG Binding Protein 2 (Mecp2) in vivo. This suppresses bystander editing very robustly, resulting in restored MeCP2 function in the CNS of Rett syndrome mouse models, and is an illustrative example of major advances attained by controlling on-target activity [47].

Substantial progress has also been made in the field of RNA targeting and editing using CRISPR–Cas13 systems, from guide RNA design to effector development. A systematic comparison of chemically modified crRNAs at different sites has shown that crRNA chemistry and resulting Cas13-mediated gene knockdown efficiency and stability could be significantly enhanced for human cell lines as well as primary T cells suggesting that chemical modification of guide RNAs is a viable approach to improve the activity of Cas13 [48]. In RNA-BE applications, comparative analyses of Cas13-ADAR editors derived from PspCas13b and Cas13bt3 alongside AAV-delivery in mouse models expressing pathogenic USH2A mutations verified that transcript-targeting Cas13 RNA editing functionally reverses disease-causing mutations in inherited retinal degeneration [49]. The compact Cas13bt3 was used to construct a single-vector AAV RNA therapeutic system and specific, efficient silencing of Vascular Endothelial Growth Factor A (VEGFA) mRNA was observed in human retinal organoids and VEGFA transgenic mice. The validation of its efficacy in models of retinal neovascular disease was a proof-of-concept that anti-VEGF RNA medicine approaches are possible and worthy candidates for in vivo RNA therapy for ocular diseases [14].

At the post-transcriptional level, a catalytically dead form of dCasRx has also been used to build programmable RNA splicing modulation systems. These systems can powerfully alter endogenous alternative splicing patterns with little impact on the total abundance of transcripts, and have been used to dissect key splicing regulatory elements, thereby enhancing our arsenal for studying and manipulating alternative splicing at the RNA level [12, 50]. Likewise, a dCas13d-based programmable RNA-binding platform, termed SpliceRUSH, was developed for high-throughput screening of proximal and distal splicing-regulatory elements (SREs) in their native sequence context. In SMN2, a therapeutically relevant target for spinal muscular atrophy, SpliceRUSH identified both known and previously unrecognized SREs, and follow-up validation showed that these elements could be functionally targeted by either dCas13d/gRNA or antisense oligonucleotides (ASOs) to modulate exon 7 splicing. These findings establish a general framework for mechanistic studies of RNA splicing regulation and support its translational relevance for RNA-targeted therapeutic development [50].

Regarding spatiotemporally applicable regulation, the development of optogenetic strategies in combination with Cas13 platforms has further advanced. Light-mediated Cas13 systems, including paCas13 and padCas13, enable low-background, highly inducible, and reversible RNA interference or A-to-I and C-to-U RNA editing in mammalian cells, with precise spatial and temporal control of disease-associated transcripts and critical signaling pathways [11]. CRISTAL consists of a library of orthogonal, inducible split-Cas13 effectors that can be precisely turned on or off using small molecules and lends itself to multi-input logic-gate design for control of multiple RNA targets in vitro and in vivo. This system provides a strong basis for spatially and temporally regulated RNA control and other Cas13-based synthetic biology tools in mammalian tissues [51]. In the broader optogenetic context, the optimized two-plasmid light-activated CRISPR effector (2pLACE) system, together with OptoPlate-96, provides tunable, reversible, and high-throughput light-inducible transcriptional control in mammalian cells. This combination offers a scalable platform for the rapid optimization of optogenetic CRISPR-based gene regulation systems [52–54].

**Fig. 1:**
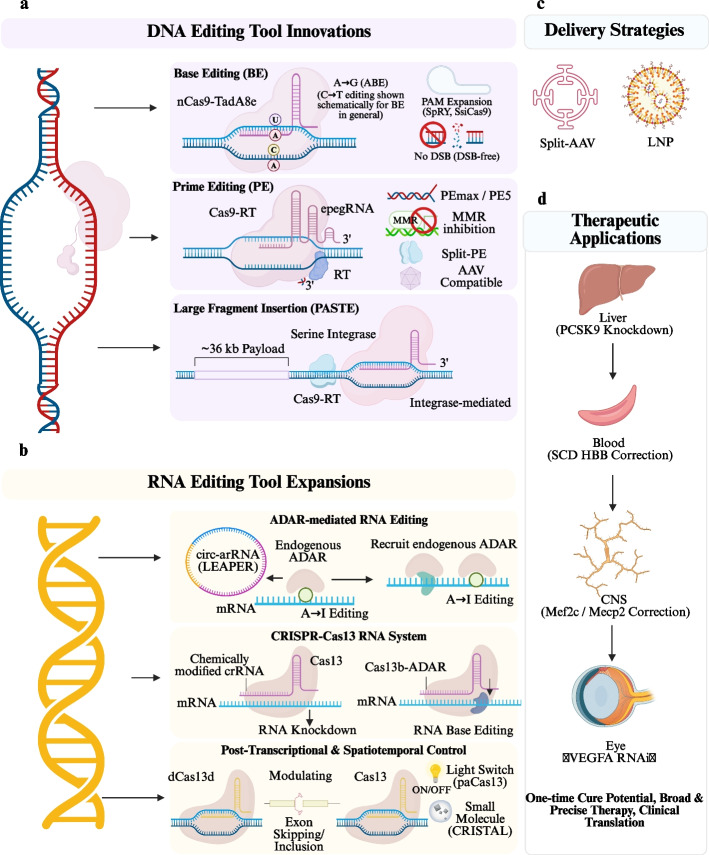
Evolution of gene-editing tools, delivery strategies, and their applications in the treatment of human diseases. **a** DNA editing tool innovations, including base editing (BE), prime editing (PE), and large-fragment insertion strategies (e.g., probabilistic Alignment of Spatial Transcriptomics Experiments [PASTE]), highlighting advances in editing precision, PAM expansion, and double-strand break (DSB)-free genome modification. **b** RNA editing tool expansions, including endogenous Adenosine Deaminase Acting on RNA (ADAR)-mediated editing (e.g., Leveraging Endogenous ADAR for Programmable Editing of RNA [LEAPER]), Clustered Regularly Interspaced Short Palindromic Repeats (CRISPR)-Cas13-based systems, and post-transcriptional and spatiotemporal regulatory approaches. **c** Representative in vivo delivery strategies, including lipid nanoparticles (LNP) and split adeno-associated virus (AAV) systems. **d** Therapeutic applications across multiple organ systems, including liver (Proprotein Convertase Subtilisin/Kexin Type 9 [PCSK9 knockdown]), hematologic diseases (sickle cell disease [SCD] HBB correction), central nervous system (CNS) disorders (Mef2c/Methyl-CpG Binding Protein 2 [Mecp2 correction]), and ophthalmic diseases (Vascular Endothelial Growth Factor A [VEGFA] RNA interference). Abbreviations: AAV, adeno-associated virus; ADAR, Adenosine Deaminase Acting on RNA; BE, base editing; CRISPR, Clustered Regularly Interspaced Short Palindromic Repeats; CNS, central nervous system; DSBs, double-strand breaks; LNP, lipid nanoparticle; LEAPER, Leveraging Endogenous ADAR for Programmable Editing of RNA; Mecp2, Methyl-CpG Binding Protein 2; Mef2c, Myocyte Enhancer Factor 2 C; PASTE, probabilistic Alignment of Spatial Transcriptomics Experiments; PCSK9, Proprotein Convertase Subtilisin/Kexin Type 9; PE, prime editing; SCD, sickle cell disease; VEGFA, Vascular Endothelial Growth Factor A

In summary, RNA editing tools are moving from early proof-of-concept experiments toward a rich technological landscape that has significant in vivo applicability. Ranging from endogenous ADAR-recruitment-dependent BE platforms, Cas13-mediated transcript editing, splicing modulation and reversible interference, to optogenetic and small-molecule inducible systems with exquisite time resolution for precise control in vivo, RNA editing is rapidly pushing the limits of what is achievable post-transcriptionally. Although such non-permanent intervention is expected to lower genetic risks, it will also require more efficient editing, durability, and delivery. Whether RNA editing can in the end succeed in therapeutic translation will depend on the correlated improvement of specificity control, in vivo delivery, and regulatory precision. Figure 1 profiles the general evolutionary trend of gene editing techniques and their translational course toward the clinic in recent years, where new architectures for DNA/RNA editing tools are depicted as well as delivery modalities and a representative selection of therapeutic uses.

### Comparative analysis of DNA editing and RNA editing

**Fig. 2:**
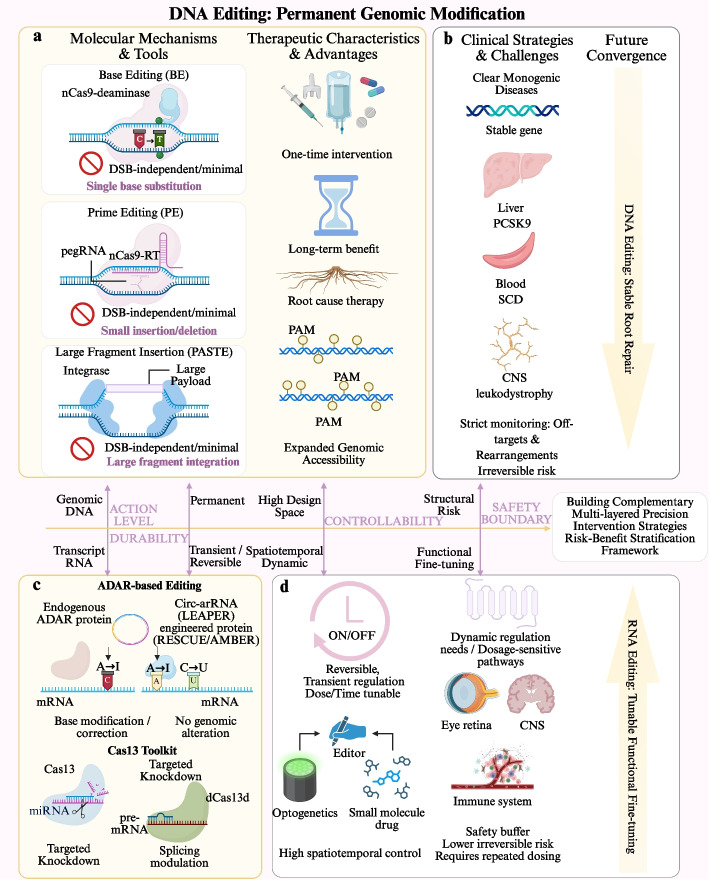
Multidimensional comparison of DNA editing and RNA editing in molecular mechanisms, therapeutic characteristics, and clinical translation logic. **a** DNA editing strategies, including base editing (BE), prime editing (PE), and large-fragment insertion via probabilistic Alignment of Spatial Transcriptomics Experiments (PASTE), enable double-strand break (DSB)-independent genome modification, supporting precise and durable genetic correction. **b** Clinical applications of DNA editing in monogenic diseases across major organs, including liver (liver-Proprotein Convertase Subtilisin/Kexin Type 9 [PCSK9]), blood (sickle cell disease [SCD]), and central nervous system (central nervous system [CNS]; leukodystrophy), alongside challenges such as off-target effects, genomic rearrangements, and safety concerns associated with permanent genome modification. **c** RNA editing approaches based on Adenosine Deaminase Acting on RNA (ADAR) systems and Clustered Regularly Interspaced Short Palindromic Repeats (CRISPR)–Cas13 enable transcript-level modification, including base editing, targeted knockdown, and splicing regulation, without altering genomic DNA. **d** RNA editing enables reversible and tunable regulation with spatiotemporal control (e.g., optogenetics and small molecules), offering improved safety but typically requiring repeated administration. The mid-line indicates a continuum of the two technologies not only in the level scavenging and persistence as well as their tolerance margins but also embedded in subsequent emergent expansion to disease selection and future multilayered precision intervention strategies. Abbreviations: ADAR, Adenosine Deaminase Acting on RNA; BE, base editing; CNS, central nervous system; CRISPR, Clustered Regularly Interspaced Short Palindromic Repeats; DSB, double-strand break; PASTE, probabilistic Alignment of Spatial Transcriptomics Experiments; PCSK9, Proprotein Convertase Subtilisin/Kexin Type 9; PE, prime editing; SCD, sickle cell disease

Existing gene-editing technologies have evolved into a dual-direction system in which DNA editing and RNA editing are advancing side by side. The two approaches differ markedly in molecular mechanism, intervention durability, safety-risk profile, and clinical feasibility. DNA editing aims at permanent genomic modification and is therefore often considered for one-time treatment with durable benefit, whereas RNA editing acts at the post-transcriptional level and offers reversibility together with potential spatiotemporal control [13, 55]. For a broad comparison of DNA and RNA editing in terms of technical principles, clinical characteristics, and application prospects, Fig. 2 systematically illustrates both classes of technologies, including their molecular mechanisms, therapeutic advantages, safety boundaries, and future development trends.

DNA editing technologies, including BE and PE, permit multiplexed genome rewritings from SNVs to small indels, medium insertions/deletions, and even extensive fragment integrations without or with very limited dependence on DSBs [2, 22, 24, 26]. Emerging data from in vivo disease model studies in hematopoietic, liver and CNS diseases, as well as metabolic leukodystrophies, have confirmed long-term correction of pathogenic mutations with sustained phenotypic reversal, highlighting DNA editing as a root-cause therapeutic approach characterized by “one-time intervention with long-term benefit” [26–29]. Meanwhile, expansion of PAM recognition spectra and generation of Cas9 variants with enhanced genome accessibility continue to broaden design flexibility, supporting tailor-made interventions in genetically complex disease settings [30, 32, 33].

RNA-based methods have intrinsic merits of reversibility and potential spatiotemporal precision. Recent engineered A-to-I and C-to-U RNA editing platforms, including RESCUE, LEAPER, AMBER, and ProAPOBECs, achieve fine-tuned base-level editing or functional repair of disease-causing transcripts without altering the underlying genomic sequence [13, 39, 42, 46, 56]. Such an approach minimizes the possibility of irreversible genetic changes while retaining the option to modulate dose or treatment duration according to disease progression and treatment-associated toxicity. Concomitantly, Cas13-based RNA targeting and editing systems have become relatively comprehensive engineering platforms, encompassing guide RNA chemical modification, compact effector optimization, and AAV-compatible single-vector packaging strategies. These tools have achieved in vivo success with transcript-specific silencing or RNA base editing in models of retinal neovascularization and inherited retinal degeneration [14, 48, 49, 57]. Expanding on this insight, dCasRx/dCas13d systems integrate programmed control into RNA splicing networks, while optogenetic and small-molecule–dependent approaches (e.g., paCas13, CRISTAL, 2pLACE) enable greatly improved temporal and spatial precision over RNA editing and regulation.

Notwithstanding the significant advances in editing accuracy, tool repertoire and efficiency with in vivo delivery, the clinical translation of both DNA editing technologies and RNA editing technologies is hindered by numerous hurdles. Although strategies for low- or no-DSB editing minimize classical genome damage, the long-term in vivo effect of bystander editing and putative structural variations has not been systematically assessed. Furthermore, many of the current in-vivo studies strongly depend on particular delivery systems and experimental setups; leading to doubts whether similar results can be expected when applying them to other types of tissue and diseases. RNA editing is superior in this regard due to reversibility, yet there remain fundamental problems with the efficiency of editing, how long the effect lasts and whether a dosing strategy could be established that would enhance therapeutic stability.

Importantly, RNA editing should not be regarded merely as a transient alternative to DNA editing, but rather as a strategically distinct therapeutic modality, particularly suited for disease contexts requiring reversibility, dosage flexibility, or enhanced safety margins.

## Current status of gene editing in disease therapy

Gene editing technologies, encompassing both DNA and RNA editing, are reshaping therapeutic development for a broad range of previously intractable disorders. Notably, genome editing has already reached clinical application in monogenic blood diseases, while both DNA- and RNA-targeting platforms are generating expanding preclinical and translational evidence in cancer, inherited disorders, and chronic or infectious diseases. Moreover, the key challenges facing these technologies at present are also examined, providing an intuitive index to assess the clinical translational prospect of gene editing technologies and guide their future directions.

### Current status of gene-editing-based therapies for genetic diseases

Mendelian disorders have become one of the first therapeutic areas to be systematically tested in clinical trials for genome editing approaches. Well-defined causative genes, relatively simple underlying molecular pathogenesis and measurable clinical end points provide an attractive framework in which to assess the benefit/risk of genome-edited strategies. Progress in ex vivo editing, delivery systems and second-generation editing tools have rapidly advanced genome editing from preclinical investigation into clinical trials for various monogenic diseases, with some showing therapeutic effects approaching a functional cure. This section highlights clinical advances and selected preclinical data supporting the prospect of genome editing in a monogenic disorder across numerous organ systems.

**Fig. 3:**
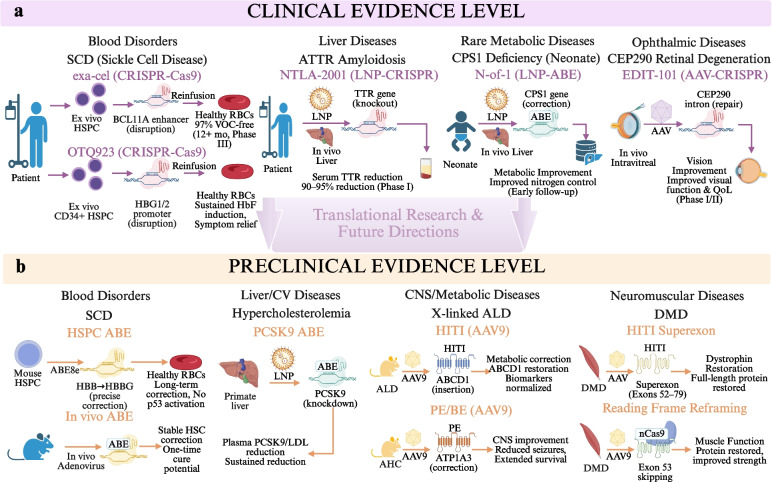
Clinical and preclinical evidence supporting gene editing in the treatment of inherited diseases. **a** The upper panel illustrates the clinical evidence level, highlighting representative applications in hematologic (e.g., sickle cell disease [SCD] treated with ex vivo Clustered Regularly Interspaced Short Palindromic Repeats [CRISPR]-Cas9-edited hematopoietic stem/progenitor cells [HSPCs]), liver (e.g., Transthyretin Amyloidosis [ATTR] via lipid nanoparticle [LNP]-mediated in vivo CRISPR), rare metabolic (e.g., Carbamoyl Phosphate Synthetase 1 [CPS1] deficiency via LNP-delivered Adenine Base Editor [ABE]), and ophthalmic diseases (e.g., Centrosomal Protein 290 kDa [CEP290] via adeno-associated virus [AAV]-based CRISPR), demonstrating gene disruption or correction with clinically relevant outcomes and reflecting the advanced stage of clinical translation. **b** The lower panel summarizes the preclinical evidence level across blood, liver/cardiovascular, central nervous system (CNS0/metabolic, and neuromuscular diseases, using gene-editing approaches such as ABE, prime editing 9PE), and homology-independent targeted integration [HITI] delivered via viral (e.g., AAV9) or non-viral systems. These studies demonstrate gene correction, biomarker normalization, functional recovery, and therapeutic potential, representing earlier-stage validation that underpins subsequent clinical development. Abbreviations: AAV, adeno-associated virus; ABE, Adenine Base Editor; ATTR, Transthyretin Amyloidosis; CEP290, Centrosomal Protein 290 kDa; CNS, central nervous system; CPS1, Carbamoyl Phosphate Synthetase 1; CRISPR, Clustered Regularly Interspaced Short Palindromic Repeats; HITI, homology-independent targeted integration; HSPCs, hematopoietic stem/progenitor cells; LNP, lipid nanoparticle; PE, prime editing; SCD, sickle cell disease

Hematopoietic monogenetic disorders were some of the earliest in which clear clinical benefit was established. In a phase III, single-arm, open-label trial, autologous hematopoietic stem and progenitor cells edited with CRISPR-Cas9 ex vivo at the erythroid-specific enhancer of B-cell leukemia/lymphoma 11 A (BCL11A) were reinfused as exagamglogene autotemcel, and 97% of patients were free from severe vaso-occlusive crises for at least 12 months after a median follow-up of 19.3 months. Safety profile was largely similar to conventional myeloablative conditioning and autologous HSC transplantation [58]. Likewise, CRISPR-Cas9-induced disruption of the Hemoglobin Subunit Gamma (HBG) 1/HBG2 promoters with OTQ923 in human CD34⁺ HSPCs to reactivate fetal-type hemoglobin (HbF) expression also demonstrated durable and broad induction of HbF levels within patients with severe SCD that correlated with significant improvement of clinical parameters, further validating that genome-editing-based induction/re-expression of HbF is a feasible approach [59]. In preclinical experiments, the rapid, high-activity ABE8e-NRCH safely and effectively converted disease causing HBBS alleles to nonpathogenic HBBG at therapeutic levels in patient-derived HSPCs and humanized mouse models of SCD. Extended engraftment substantially ameliorated sickling and associated pathologies of erythrocytes without detectable Cas9 nuclease-mediated p53 activation or large deletions in the genome (altogether supportive of long-term correction) establishing proof-of-principle for one-time, autologous base-editing therapy for SCD [26]. Extending the strategy of in situ BE, a single intravenous or subcutaneous administration of a non-integrative adenoviral vector allowed for in vivo BE of HSCs in SCD mice targeting specifically the G-Makassar variant—without detectable off-targeting—and achieved stable correction at the biological level. These data indicate in vivo BE of HSCs may be a scalable cure, like one-time administration [60]. Figure 3 presents a joint representation of the steady progression from preclinical validation into clinical implementation of genome-editing technologies through critical clinical findings and representative preclinical milestones across organ systems.

In addition to hematologic diseases, in vivo genome-editing control has now become a significant consideration for monogenic metabolic disorders and liver-based genetic diseases. A phase I clinical trial found that a single dose of Intellia Therapeutics, Inc. (NTLA)−2001, the LNP–encapsulated CRISPR-Cas9 therapeutic, which was delivered only once through intravenous infusion in patients with hereditary Transthyretin (TTR) amyloidosis, resulted in safe and durable TTR knockout and yielded fluctuations that resulted in a mean decrease of serum TTR of 50% to 90%. This is a precedent-setting clinical demonstration of concept for in vivo genome-editing and treatment of monogenic diseases [61]. Delivery of LNP, mRNA encoding for ABE to liver in nonhuman primates led to low and moderate/high editing efficiencies and reductions of plasma PCSK9 and LDL cholesterol levels that were sustained over time, which highlights the potential feasibility of in vivo BE treatment of liver-associated monogenic diseases and inherited cardiovascular endocrine risk factors [62]. A neonate with severe carbamoyl-phosphate synthetase 1 deficiency was treated with personalized LNP–delivered in vivo BE within the framework of an individualized N-of-1 therapy. Early follow-up at 2 infusions indicated improved control of nitrogen metabolism without serious adverse events, and this case series provides encouraging preliminary clinical support for personalized genome-editing approaches in rare monogenic metabolic diseases [63]. In ALD patient-derived and mouse fibroblasts, codelivery of BE with HITI carried out by in vivo gene insertion using an AAV9 vector efficiently corrected ABCD1 expression and normalized the levels of very long chain fatty acid-related biomarkers. These findings suggest that HITI-mediated restoration of mutant genes could be used as a promising form of therapy for ALD and other X-linked monogenic diseases [29].

Early indications of in vivo genome-editing in inherited ocular and CNS disorders have likewise reached the translational orbit. Intravitreal injection of the gene-editing strategy EDIT-101 in a phase 1/2 trial for patients with inherited retinal degeneration caused by Centrosomal Protein 290 kDa (CEP290) resulted in good tolerability of in vivo CRISPR-Cas9 editing and improvements in visual function, as well as vision-related quality of life, observed in some participants. This is consistent with in vivo genome-editing being a potentially therapeutic intervention for inherited retinal diseases [64]. In vivo, targeted correction of pathogenic mutations by PE 2/3 delivered either hydrodynamically or with AAV in mouse models of hereditary tyrosinemia and LCA patient derivatives significantly ameliorated disease phenotypes without measurable off-target activity, demonstrating an essential platform for in vivo genetic mutation correction by PE [65]. In the context of a study on alternating hemiplegia of childhood (AHC), both BE and PE efficiently repaired disease-causing ATP1A3/Atp1a3 mutations in patient-derived cells and two AHC mouse models. Further, AAV9 mediated in vivo CNS editing significantly ameliorated seizure-like episodes, motor and cognitive deficits as well as an increased in life span suggesting that one-time PE offers promise as a causative therapy for inherited neurological diseases [66].

In the field of neuromuscular genetic diseases, Duchenne muscular dystrophy (DMD) is an important paradigm disease for large fragment gene insertion and in-frame correction approaches. In humanized mouse models of DMD, AAV-mediated delivery of CRISPR-Cas9 and an insert DNA containing sequences homologous to the DMD target site successfully achieved HITI-mediated precise insertion of the exon 52 that is missing or a “superexon” encompassing the terminal 28 exons in between DMD introns, thereby reinstating full-length dystrophin expression both in the skeletal and cardiac muscles [67], indicating the therapeutic impact of HITI-based large-fragment insertion for treating a variety of DMD mutations. In patient-specific iPS cells and in the generation of new humanized mouse models for exon 52 deletions, AAV9-delivered single-strand SpCas9-LVRQR-mediated exon 53 reading frame to restore dystrophin expression among tissues and sufficiently ameliorate muscle strength and histopathological abnormalities, serving as an optimized in vivo approach for correcting DMD [68]. In total, clinical and preclinical studies share accumulated knowledge on the ability of CRISPR-Cas9, BE, PE and HITI to revolutionize therapeutic treatments for a variety of monogenic diseases in a manner that paves the way for future safety assessments and long-term monitoring.

Several approaches have confirmed the feasibility of, and potential durability in, repairing the causal mutation across a variety of organ systems from ex vivo editing HSPCs for hematologic monogenic diseases to in vivo liver, retina, and CNS editing using LNP or AAV vectors. The advent of BE, PE and HITI is extending the treatment spectrum for mutational types, gene structure complexity DSBs and mitigates some risks further. In this way genome editing will move beyond symptom-treating interventions to one-and-done treatments targeting the molecular etiology of disease, providing a feasible vision for curative treatment of monogenic genetic diseases.

### Applications and challenges in cancer therapy

**Fig. 4:**
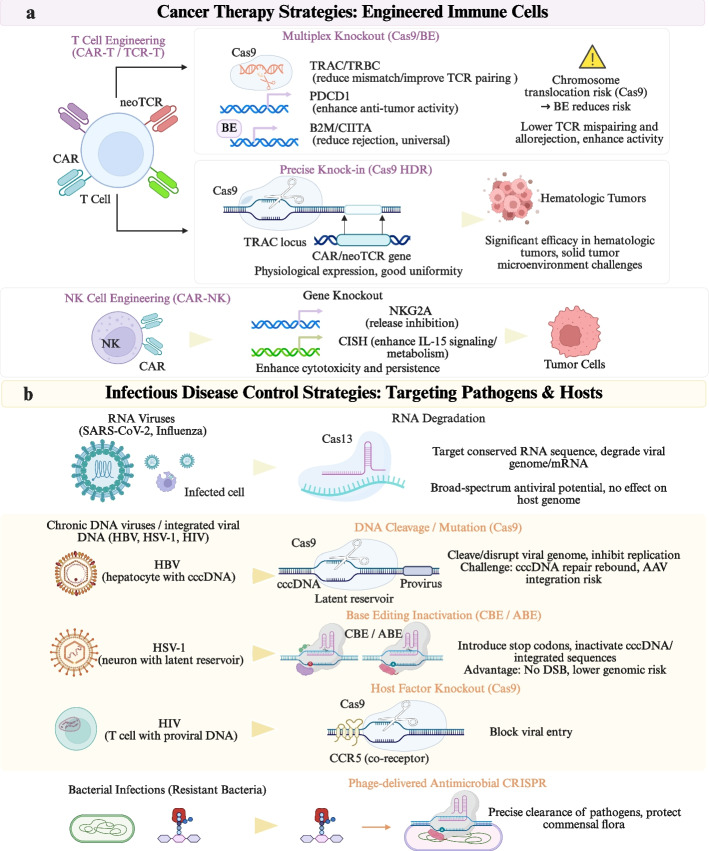
Applications of Gene Editing in the Precision Management of Cancer and Infectious Diseases Using Engineered Immune Cells. **a** Cancer therapy strategies using engineered immune cells. T cell engineering (Chimeric Antigen Receptor T-cell therapy [CAR-T]/TCR-T) employs multiplex gene knockout (e.g., T Cell Receptor Alpha Constant region [TRAC]/T Cell Receptor Beta Constant region [TRBC], PDCD1, β2-microglobulin [B2M]/Class II Transactivator [CIITA]) via Cas9 or base editing to reduce mispairing and rejection while enhancing anti-tumor activity and persistence. Targeted knock-in at the TRAC locus enables controlled CAR expression and improved uniformity. NK cell engineering (CAR-NK) further enhances cytotoxicity and metabolic fitness through gene knockout (e.g., NKG2A, Cytokine-Inducible SH2-Containing Protein [CISH]). These approaches improve efficacy in hematologic malignancies while highlighting challenges in solid tumors and genome-editing-associated risks. **b** Infectious disease control strategies targeting pathogens and host factors. RNA-targeting systems (Cas13) degrade viral RNA (e.g., Severe Acute Respiratory Syndrome Coronavirus 2 [SARS-CoV-2], influenza) without altering the host genome. DNA-targeting approaches (Cas9) disrupt viral genomes (e.g., Hepatitis B virus [HBV] covalently closed circular DNA [cccDNA], Herpes Simplex Virus type 1 [HSV-1]) or inactivate them via base editing, and host factor knockout (e.g., CCR5 in HIV) blocks viral entry. Phage-delivered Clustered Regularly Interspaced Short Palindromic Repeats [CRISPR] enables precise clearance of pathogenic bacteria while preserving commensal microbiota. Abbreviations: B2M, β2-microglobulin; CAR-T, Chimeric Antigen Receptor T-cell therapy; CISH, Cytokine-Inducible SH2-Containing Protein; CIITA, Class II Transactivator; CRISPR, Clustered Regularly Interspaced Short Palindromic Repeats; HBV, Hepatitis B virus; HSV-1, Herpes Simplex Virus type 1; SARS-CoV-2, Severe Acute Respiratory Syndrome Coronavirus 2; TRAC, T Cell Receptor Alpha Constant region; TRBC, T Cell Receptor Beta Constant region; cccDNA, covalently closed circular DNA

Thanks to the ongoing evolution of genome-editing tools, in particular coordinated multi-locus engineering and controllability in engineered immune cell therapies are evolving from “proof-of-concept” towards iterative clinical products, increasingly solidifying their technological identity within precision oncology. Multiplex editing of receptor architecture, signaling circuitry, and immune regulator nodes could offer rational modification strategies for the improvement in function as well as reactivity in clinical application. The corresponding strategies for immune cell engineering and their application principles in various types of cancers are presented in Fig. 4.

CRISPR–Cas9-based ex vivo engineering serves as a platform to simultaneously target receptor expression and immune-suppressive pathways [69–71]. In human T cells, targeting of the T Cell Receptor Alpha Constant region (TRAC)/T Cell Receptor Beta Constant region (TRBC) to minimize TCR mispairing and overexpression of an NY-ESO-1–specific TCR, combined with deletion of PDCD1 to increase antitumor efficacy, has been shown to be feasible and safe in phase I trials for treatment-refractory cancers; edited cells engrafted in vivo for months. Chromosomal translocations were identifiable but their incidence diminished with time, supporting clinical progression, and highlighting the requirement for continued genomic cautionary editing [72]. Solid tumor-directed efforts are also tracking well, mesothelin-specific Chimeric Antigen Receptor T-cell therapy (CAR-T) cells with dual PD-1 and TCR knockout exhibited manageable safety and feasibility in a phase I dose escalation trial of patients with mesothelin positive solid tumors, however, response rates were modest; both the immunosuppressive environment within the tumor bed and poor in vivo cell persistence are likely major bottlenecks [73].

In terms of manufacturing and product-related issues, non-viral and clinical grade approaches are emerging as key. In tumor-infiltrating lymphocyte (TIL) expansion and reinfusion systems, a non-viral method that does not require plasmid has been incorporated for PD-1 knockout and can achieve 87.5% downregulation of PD-1 after expansion in multiple TILs derived from solid cancers without detectable off-target effects or compromised proliferation, providing practical evidence to support Good Manufacturing Practice (GMP)-compliant enhancement of functionality [74]. Non-viral precision editing has also been applied to personalized targeting strategies. NeoTCR-T cells, generated by knocking in patient-specific neoantigen-reactive TCRs at the TRAC locus while simultaneously disrupting endogenous TRAC/TRBC, have successfully completed Phase I clinical trials. These engineered T cells can traffic into tumor tissues; however, clinical outcomes have largely manifested as stable disease or progression, accompanied by immune-related adverse events. These findings indicate that further improvements in therapeutic efficacy will depend on optimized target antigen selection, modulation of the tumor microenvironment, and enhancement of cellular persistence [75]. Employing the same anchor locus, Cas9-assisted HDR allows virus-free CAR knock-in at the TRAC locus; gene-targeting efficiency and cell recovery can be improved by pharmacologic inhibition of DNA sensing and enhancement of HDR. The generated TRAC-incorporated CD19-CAR-T cells mediate antigen-specific lysis in vitro and prolong survival and slow leukemia progression in xenografted leukemia models in vivo, while showing reduced cleavage at assessed potential off-target sites [76, 77]. Toward off-the-shelf products, multiplex editing on non-viral platforms could efficiently knock out β2-microglobulin (B2M), TRAC and PD-1 while integrating Disialoganglioside 2 (GD2)-CAR in the primary T cells, generating candidate allogeneic CAR-T cells with central memory phenotype, high cytotoxicity to GD2-positive neuroblastoma cells and less chromosomal abnormality as well as off-target effects [78].

To reduce the risk of DNA DSBs and enable efficient multiplex genome engineering, BEs are increasingly adopted for multi-gene inactivation [79–81]. Mutual sequential knockout of TCR/CD3 and CD7 performed during the generation of CAR-T product prevents “T-cell fratricide” and provides specific cytotoxicity to T-ALL in vitro and in vivo with reduced genomic risks including chromosomal translocations as compared to Cas9 nuclease-mediated editing [82]. Universal CD7-targeted CAR7 T cells simultaneously inactivated many genes without generating DNA breaks and induced a rapid molecular remission in a phase I study of relapsed/refractory childhood T-ALL, albeit with some toxicities including cytokine release syndrome, myelosuppression, and predisposition to opportunistic infections suggesting that the potential clinical benefit must be weighed against immune-related risks [83]. In a four locus multiplex model (TRAC/CD3E, B2M, Class II Transactivator [CIITA], Poliovirus Receptor [PVR]), adenine BE for the production of allogeneic CAR-T cells resulted in enhanced outgrowth and tractable off target profiles, without any evidence of chromosomal translocations nor activation of p53/DNA damage response pathways, as well as superior antitumor activity and survival advantage in leukemia models, thereby providing proof-of-concept for high throughput multi-locus engineering [80]. In addition to T cells, genome-editing approaches have also been used to augment the function and persistence of NK cells. KLRC1 knockout overcomes HLA-E–mediated inhibition, thereby enhancing the cytotoxicity of CD33-targeted CAR-NK cells against AML cell lines and primary blasts in vitro and in vivo, suggesting the added value of combining immune-checkpoint editing with cell therapy [84, 85]. On the other hand, deletion of Cytokine-Inducible SH2-Containing Protein (CISH) in human induced pluripotent stem cell (iPSC)-derived NK cells promoted IL-15–mediated JAK–STAT responses, enhances mTOR-associated metabolic adaptation, and thus results in more efficient expansion and a wide-reaching low-cytokine condition antitumor effect of higher magnitude, as well as increased persistence and efficacy against leukemia xenograft models [86].

Moreover, genomic stability and manufacturing controllability are still bottlenecks. Repeated cutting at targeted genomic sites by Cas9 can induce structural variation including chromosomal translocations; fusion of Cas9 to TREX2 (Cas9TX) abolishes re-cutting, decreases large deletions and translocations, nearly eliminates detrimental translocation events during multiplexed CAR-T editing—providing a widely applicable engineering strategy to enhance the safety of genome editing in cellular therapeutics [87]. Based on existing evidence, immunosuppression in solid-tumor microenvironments, insufficient in vivo persistence, immune-related toxicities and danger of structural variation hamper indication expansion and general efficacy [88–90].

In brief, genome editing has led cancer cell therapeutics to move to an engineering mode through addressing multiple targets in a coordinated fashion. Receptor reconfiguration and immune checkpoint ablation via Cas9, as well as base-editing–enabled multi-gene inactivation in the absence of DNA DSBs, have shown a definite functional benefit in hematologic malignancies and paved the way for universal CAR-T and CAR-NK products. However, factors such as the immune suppressive tumor microenvironment, restricted cellular persistence and immune‐related toxicities restrict the translation of efficacy, whereas risks associated with structural variants and cell manufacturing standardization represent remaining barriers concerning safety and industrialization. The advancement of editing accuracy, parallel optimization of delivery and manufacturing systems, and monitoring of genome integrity will ultimately dictate how broad the clinical application range of engineered immune therapies can be.

### Gene-editing strategies for infectious diseases

As DNA- and RNA-based gene-editing tools are constantly improved for nucleic-acid-specificity, delivery mechanisms, and in vivo safety, the application of these systems against infectious diseases is moving from inhibition of individual pathogens toward targeted disruption of essential steps in viral and bacterial life cycles. DNA- and RNA-based gene-editing interventions will therefore move away from “programmable recognition of pathogen nucleic acids” toward multipronged approaches for host, viral reservoir and targeted depletion of the pathogen populations.

For RNA viruses, the PAC-MAN method uses CRISPR–Cas13 to target and destroy conserved RNA sequences of Severe Acute Respiratory Syndrome Coronavirus 2 (SARS-CoV-2) and influenza A virus in human lung epithelial cells, leading to a striking reduction in viral load. A small number of crRNAs can also target multiple coronavirus sequences, highlighting the prospect of pan-coronavirus activity [91]. Cas13d-based systems can inhibit multiple SARS-CoV-2 variants and a range of human coronaviruses in vitro, while when combined with LNP delivery, effectively lower viral titers—Omicron included—in primary airway epithelial ALI models. These results further advocate for the development of nucleic-acid–based targeting, in addition to vaccines and small molecule antivirals [92].

Editing modalities for CHB mainly concentrate on suppressing and inactivating cccDNA [93]. Causey et al. have shown that AAV-mediated delivery of HBV-specific SaCas9 under entecavir could accomplish intrahepatic editing and trended towards lower intra-hepatic HBV DNA/cccDNA levels with an acceptable safety profile, providing the first indications for the potential application of in vivo CRISPR as a curative step [94]. Although CRISPR-Cas9 mediated cleavage of cccDNA can inhibit its replication, the double gRNA-mediated cleavage/repair could produce circular “post-editing replication-/transcription-competent” HBV episomal DNA which prompts a requirement for monitoring and mitigating the risk of such variants [95]. Additionally, cccDNA silencing itself can rebound as a consequence of rcDNA-mediated recycling; prior depletion of rcDNA by the time of Cas9 RNP administration may dampen cccDNA resupply and aid in infection clearance, thereby providing a rationale for an anti-HBV functional-cure strategy based on short-lived RNPs together with reverse transcriptase inhibitors [96–98]. For the DSB-independent alternatives, CBEs can introduce in-frame stop codons to cccDNA and host integrated HBV sequences, leading to long term inhibition of replication and decreased Hepatitis B surface antigen (HBsAg). In a mouse model, LNP delivery in HBV minicircle resulted in a significant reduction of serum HBV DNA (> 3 log10) and HBsAg level (> 2 log10), with most mice achieving HBsAg seroclearance [99]. Conversely, Cas13b can be programmed to cleave HBV pregenomic RNA and viral mRNAs, resulting in inhibition of viral replication and protein expression in cells, as well as reductions in HBsAg levels in vivo, highlighting the potential utility of RNA-targeting antiviral strategies [100].

Latency and recurrent outbreaks also make HSV-1 a target for editing strategies with latent reservoirs becoming the focus of increasing attention [101]. HELP directly cuts the viral genome to inactivate replication and cure herpetic stromal keratitis in multiple mouse models (and can be retrogradely transported from corneal cells to treat trigeminal ganglion latent reservoirs), and is safe and effective against HSV by direct application on human corneal explants and in latently infected mice [102]. In a single-center, open-label clinical trial study, a single local injection of the HSV-1 targeting CRISPR delivery during corneal transplantation in three patients with therapy-refractory HSV-1 stromal keratitis was without detectable GUIDE-seq off-target cleavage or systemic adverse events and led to sustained viral negativity with a mean follow-up of 18 months, demonstrating acceptable preliminary safety and clinical feasibility [103]. Additionally, single-dose, combined AAV9 delivery of SaCas9 and dual gRNAs targeting the essential HSV-1 genes ICP0/ICP27 led to repression of reactivation and shedding in a latent rabbit keratitis model as well as decreased levels of viral DNA/RNA in trigeminal ganglia, supporting the therapeutic positioning aimed at “reducing recurrence risk” [104]. Regarding HIV, the focus in editing approaches is on simultaneous decrease of replication potential and reservoir size without inducing more immune damage [105, 106]. In HIV-1 humanized mice tolerized with long-acting antiretroviral therapy, dual-target CRISPR-Cas9 editing of CCR5 and proviral DNA resulted in clearance of replication-competence virus in 58% of animals, reconstitution of CD4^+^ T cells without evidence for off-target toxicity and serves as evidence that a combination-editing approach will be required to advance a functional cure [107]. One intravenous infusion of the AAV9-based vector EBT-001 carrying SaCas9 and dual gRNAs targeting SIV Long Terminal Repeat (LTR) and Group-specific antigen (Gag), was able to produce reservoir-wide coverage as well as proviral DNA editing in ART-suppressed rhesus macaques, without any apparent off-target or pathogenic abnormalities, thus supporting translational safety and feasibility [108].

For bacterial infections, editing tools focus on “specific eradication of pathogenic bacteria without perturbation of commensal microbiota,” where phage mediated delivery is identified as an important enabler. SNIPR002 is a precision antimicrobial candidate and its selectivity allows for it to suppress the burden of Escherichia coli in the murine gastrointestinal tract while also preventing selection for tolerant strains; SNIPR002 is well tolerated by mice [109]. Consistently, P1-derived phage vectors for chromosome-targeting CRISPR-Cas9 cause sequence-specific killing of Shigella flexneri in a zebrafish larval infection model reducing the bacterial burden and improving host survival, further supporting the potential for “phage delivery + CRISPR antimicrobials” as a reprogrammable anti-infective approach [110].

In the context of a therapeutic paradigm, gene-editing technologies are shifting the focus for intervention against infectious disease from passive retardation of pathogen spread to active modification of replicative potential and survival ecology. By tailoring viral genomes, transcripts and their latent reservoirs into desired states, the persistence of infection becomes a modifiable target itself; at the same time, phage-delivered editing systems offer molecularly precise elimination of pathogens while maintaining microbiome. While the challenges of vaccine delivery coverage, escape variants, and safety limits remain, this data-driven approach to interventions has potential as a solution for chronic and drug-resistant infections beyond traditional pharmacologic paradigms.

### Frontiers in NDs and chronic disorders

**Fig. 5:**
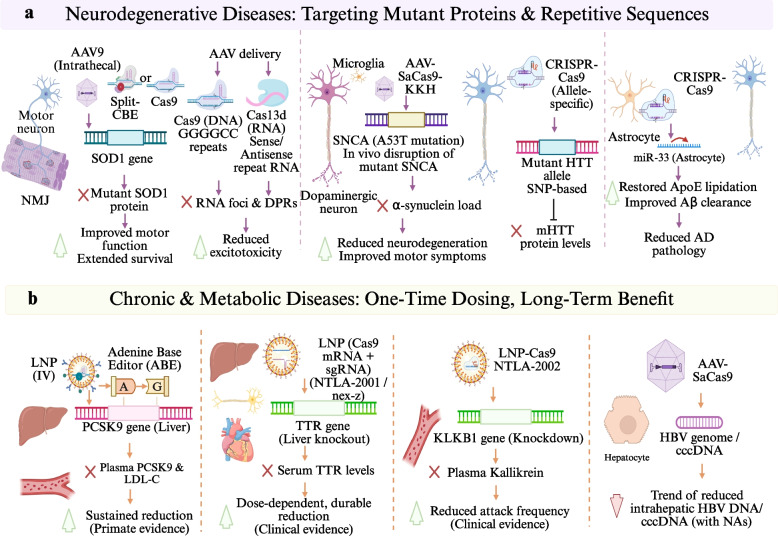
Interventional strategies and translational progress of gene editing in neurodegenerative and chronic diseases. **a** Gene-editing approaches targeting mutant proteins and repetitive sequences in neurodegenerative diseases. Adeno-associated virus [AAV]-mediated delivery enables in vivo editing in neurons and glia, including disruption of mutant genes (e.g., Superoxide Dismutase 1 [SOD1]), targeting repeat expansions at the DNA (Cas9) or RNA level (Cas13), allele-specific editing (e.g., Huntingtin [HTT]), and Synuclein Alpha [SNCA] mutation correction to reduce α-synuclein burden. Astrocyte-directed modulation further improves lipid metabolism and amyloid-β clearance. These strategies reduce neurotoxicity and improve functional outcomes. **b** Gene-editing strategies for chronic and metabolic diseases characterized by one-time intervention with durable effects. In vivo editing via lipid nanoparticle [LNP] or AAV targets liver-expressed genes, including base editing of Proprotein Convertase Subtilisin/Kexin Type 9 [PCSK9] to reduce low-density lipoprotein cholesterol [LDL-C], Cas9-mediated knockout of Transthyretin [TTR] (e.g., Intellia Therapeutics, Inc. [NTLA]−2001) and Kallikrein B1 [KLKB1] (e.g., NTLA-2002), and targeting of Hepatitis B virus [HBV] covalently closed circular DNA [cccDNA]. These approaches achieve sustained biomarker reduction and therapeutic benefit in preclinical and clinical settings. Abbreviations: AAV, adeno-associated virus; HBV, Hepatitis B virus; HTT, Huntingtin; KLKB1, Kallikrein B1; LDL-C, low-density lipoprotein cholesterol; LNP, lipid nanoparticle; NTLA, Intellia Therapeutics, Inc.; PCSK9, Proprotein Convertase Subtilisin/Kexin Type 9; SNCA, Synuclein Alpha; SOD1, Superoxide Dismutase 1; TTR, Transthyretin; cccDNA, covalently closed circular DNA

With continued improvements in CNS delivery efficiency and long-term safety of gene editing technologies, its utilization is being propelled into treatment for HDs, as well as a wide array of chronic conditions; from molecular pathology improvement to measurable functional improvements associated with lifelong therapeutic benefits. In the neurodegeneration arena, in vivo gene editing technologies has moved from correction of pathology biomarkers to evidence-based, quantitative functional improvements. This shift–from molecular correction to measurable functional benefit–and how it relates to translational vision of “single intervention, long-term benefit” in chronic diseases is summarized systematically in Fig. 5.

In a familial amyotrophic lateral sclerosis (ALS) model driven by Superoxide Dismutase 1 (SOD1) mutations, intrathecal delivery of a dual-AAV-encapsulated split CB editor, which is assembled via intein splicing, induces premature stop codons in the SOD1 gene. This strategy permanently inactivates mutant SOD1, extends survival in G93A-SOD1 mice, and alleviates muscle atrophy, denervation, and neuromuscular dysfunction [111]. Moreover, long-term delivery of sgRNAs using AAV-pseudotype (PHP) variants can establish sustained disruption of huSOD1 via CRISPR expression to protect mice from disease, including ameliorated motor performance and preservation of motor neurons and neuromuscular junctions, which extended their survival by 110–170 days [112]. For C9ORF72 GGGGCC repeat expansion related ALS and FTD, AAV-delivered CRISPR/Cas9 excisions of the expanded repeats were also reported to decrease RNA foci, dipeptide-repeat proteins in primary neurons, mouse brains, patient-derived iPSC motor neurons and brain organoids as well as alleviating major pathological aspects such as haploinsufficiency [113]. At the transcript level, a derivative of Cas13d, termed CasRx, which simultaneously targets sense and antisense repeat RNAs with a single construct, reduces both repeat transcripts and dipeptide repeat proteins and rescues excitotoxic damage in patient iPSC-derived neurons as well as two mouse models providing additional evidence for RNA-targeting strategies [114]. In Parkinson’s disease, in vivo deletion of Synuclein Alpha (SNCA) with SaCas9-KKH/sgRNA mitigates the α-synuclein burden and rescues microglial activation, nigrostriatal dopaminergic neurodegeneration, and motor deficits in an A53T-SNCA overexpressing rats [115]. In autosomal dominant neurodegenerative disorders, allele-specific CRISPR-Cas9 editing guided by common heterozygous coding SNPs enables selective inactivation of mutant Huntingtin (HTT) alleles, resulting in a marked reduction of mutant HTT protein levels [116]. In the context of Apolipoprotein E (ApoE) 4-associated sporadic Alzheimer’s disease, CRISPR/Cas9-mediated targeting of astrocytic miR-33 is associated with restoration of ApoE lipidation, improved Aβ metabolism, and attenuation of AD-like pathology, highlighting the editability and translational potential of miRNA regulatory nodes [117].

The concept of single administration, long-term benefit is even accentuated in chronic diseases. In non-human primates, a single administration of LNP-delivered CRISPR base editors potently and specifically edits PCSK9 in the liver, decreasing plasma PCSK9 by 90% and low-density lipoprotein cholesterol (LDL-C) by 60%, an effect lasting at least 8 months–demonstrating primate evidence for being able to durably control chronic cardiovascular risk [27]. On the clinical front, NTLA-2001, containing Cas9 mRNA and an sgRNA targeting TTR formulated in LNPs demonstrated dose-dependent and significant decreases in serum TTR (mean 28 day decrease of 52%–87%) in a phase I trial for Transthyretin Amyloidosis (ATTR) familial amyloid polyneuropathy with mostly mild adverse effects [61]. In an open-label phase I trial for ATTR cardiomyopathy, called nexiguran ziclumeran (Nex-z) produced durable serum TTR reductions of 89% at 28 days and 90% after a year with one single IV dose; the main safety signals were short-term infusion reactions and transient transaminase elevations [118]. Kallikrein B1 (KLKB1)-directed NTLA-2002 showed dose-dependent and persistent reduction of plasma kallikrein in a phase 1/2 study of adults with Hereditary angioedema (HAE), resulting in reductions in attack frequency along with a low incidence of serious adverse events [119]. In chronic infection, AAV-delivered SaCas9 targeting the HBV genome achieved detectable in vivo editing in humanized liver mouse models of chronic HBV infection and, on a background of nucleos(t)ide analogue therapy, showed a trend toward reduced intrahepatic HBV DNA/cccDNA with good tolerability—providing in vivo support for curative strategies aimed at cccDNA [94].

Gene-based treatments for HDs and chronic diseases by genotype editing also increase both beneficial effects and risks. Single in vivo treatment can provide long-term functional improvement, but the irreversibility of genetic modification also increases the demand for selective delivery, cell-specific targeting, and ensures long-term safety. This consideration is of particular relevance for slow turnover tissues such as the CNS, where any deviation could potentially accumulate into sustained biological effects and thereby increase the vulnerability of health-risk balance. Thus, the advancement in this field will depend less on reaching the maximum editing efficiency at the top end of this hill and more on enabling synchronized progress toward optimal interrelated control of delivery, editing accuracy, and long-term monitoring—factors that will ultimately define applications of gene editing technologies in chronic and brain diseases.

## Key challenges in therapeutic applications

**Fig. 6:**
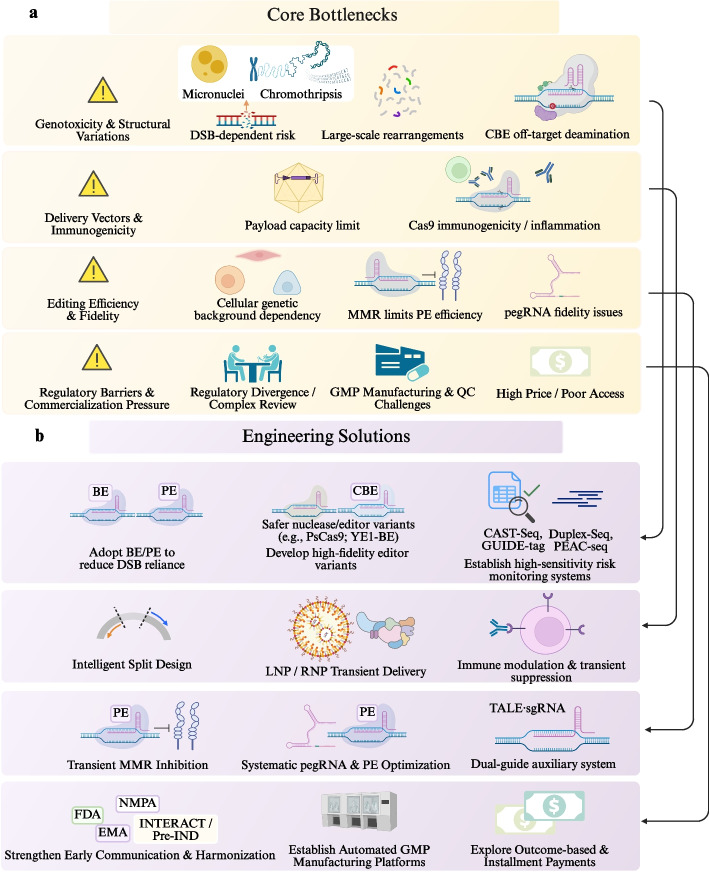
Panoramic overview of the core bottlenecks and engineering solutions for the clinical translation of gene editing. **a** Key limitations in gene-editing applications, including genotoxicity and structural variations from double-strand break [DSB]-dependent editing (e.g., micronuclei, chromothripsis, large-scale rearrangements, and off-target deamination), delivery and immunogenicity constraints (e.g., payload limits and Cas9-related immune responses), suboptimal efficiency and fidelity (e.g., cellular context dependence, mismatch repair [MMR] restriction of prime editing, and pegRNA instability), and regulatory and commercialization challenges such as complex review processes, good manufacturing practice [GMP] manufacturing barriers, and high cost. **b** Engineering strategies addressing these challenges, including DSB-independent editing (base editing [BE]/prime editing [PE]), development of high-fidelity editors and sensitive off-target detection methods (e.g., Chromosomal Aberration Sequence Tagging Sequencing [CAST-seq], GUIDE-seq, Prime Editing-Associated Chromatin sequencing [PEAC-seq]), optimized delivery systems (e.g., split designs and lipid nanoparticle [LNP]/ribonucleoprotein [RNP]-based transient delivery) with immune modulation, and enhanced editing precision via MMR inhibition, pegRNA optimization, and auxiliary guide systems. Translational barriers are further mitigated through regulatory harmonization, automated GMP platforms, and outcome-based pricing models. Abbreviations: BE, base editing; CAST-Seq, Chromosomal Aberration Sequence Tagging Sequencing; DSBs, double-strand breaks; GMP, good manufacturing practice; LNP, lipid nanoparticle; MMR, mismatch repair; PE, prime editing; PEAC-seq, Prime Editing-Associated Chromatin sequencing; RNP, ribonucleoprotein

Despite its transformative therapeutic potential, genome editing faces multi-scale barriers that hinder clinical translation. These include biological risks such as off-target activity and unintended genomic alterations, engineering limitations in delivery efficiency and tissue specificity, and broader clinical and regulatory challenges, including immunogenicity, long-term safety assessment, and ethical governance [120]. Addressing these constraints will require coordinated advances in editing precision, delivery technologies, next-generation editing modalities that avoid DSBs, and the establishment of standardized regulatory and quality control frameworks. To graphically generate an overview of this intricate translational landscape and the current solutions to obstructive elements, Fig. 6 provides a step-by-step compilation of major obstacles—from genotoxic levels and delivery vectors down to regulatory limitations and commercial scaling—along with matching responses.

### Technical limitations and existing solutions

The “challenges” discussed in this section include both technical issues that can be optimized and systemic limitations that are difficult to overcome under current conditions. Genome editing is moving from early proof-of-concept studies towards application related studies with an emphasis on in vivo delivery and clinical outcomes, this move also implies change in focus of research. On increasing the editing scale, duration of action and routes to delivery, considerations in safety and process controllability are significantly exacerbated and increasingly form a key bottleneck in translational feasibility. Right now, it would not be enough to rely on either the efficiency of editing or the precision with which cells are targeted to meet clinical needs. Thus, it is necessary to have a systematic review of the existent technical bottlenecks in the safety control, process stability and risk assessment with the search for associated technological counter-measures.

Although BEs and PEs diminish dependence on DNA DSBs in human HSPCs, the resulting undesired transcriptional responses and/or potentially genotoxic byproducts (including DSBs as well as deletions or translocations) could still occur. Remarkably, CBEs are of considerable concern under this criterion. Optimal design of mRNA structure, as well as precise timing and level of delivery expression can in part reduce such risks [121]. In DSB-mediated CRISPR–Cas9 systems, target-site cleavages can cause small insertions/deletions (indels), as well as nuclear anomalies such as micronuclei and chromatin bridges; moreover, they may lead to massive rearrangement features possibly reminiscent of a chromothripsis event. These findings suggest that SVs should be included as standard safety issues for monitoring [122]. Consistently, quantitative analyses in primary cells and cell lines show that CRISPR–Cas9 can frequently induce kilobase-scale large deletions, insertions, and local complex rearrangements. At the HBB/HBG/BCL11A loci in HSPCs, such alterations can reach approximately 11.7%–35.4%, potentially reducing the proportion of therapeutically usable alleles and affecting functional outcomes. Accordingly, these events should be treated as key endpoints in preclinical evaluation to guide protocol optimization [123].

For quantitative assessment of genotoxic risk, Chromosomal Aberration Sequence Tagging Sequencing (CAST-Seq) can be used to profile chromosomal translocations and large-scale rearrangements resulting from on-target or off-target activities in edited human CD34⁺ hematopoietic stem cells as support for pre-release risk assessment [124]. Exploiting DiSEA in combination with high-sensitivity off-target analysis by duplex sequencing, the type II-B PsCas9 subfamily shows significantly lower levels of genome-wide off-target activity and reduced chromosomal translocation risk when programmed in vitro and in cells, which may provide a path toward engineering safer nucleases for clinical applications [125]. To control off-target deamination in BE, next-generation CBEs in which the APOBEC1 deaminase source was replaced display significantly less nonspecific deamination in genomic DNA and transcriptomic RNA with on-target efficiency being preserved [126]. In addition, the “dual-guide” system composed of sgRNA and Transcription Activator-Like Effector (TALE) pooled with the low-off-target YE1 deaminase, not only effectively converts C to T but also eliminates predictable Cas9-mediated DNA off-targets in a detectable range, and can be used as a designed tool for resolving the safety problem of CBE [127].

The effectiveness and accuracy of PE depends significantly on the genetic background of cells. Screening for DNA repair factors reveals that MMR restrains the PE process, as deletion or inhibition of MMR enhances editing efficiency from 2- to 17-fold in multiple human cell types and editing contexts, providing a straightforward pathway-level target for PE improvement [128]. Limited by AAV cargo space, Rma intein sp-HA-PE delivered in dual AAV vectors allows targeted editing on endogenous loci of adult tissues (such as the mouse retina) in vivo [37]. Expanding upon this strategy, systematic optimization of PE expression, pegRNA stability and DNA repair modulation results in therapeutically relevant editing levels in a variety of mouse tissues—such as brain, liver, and heart—without detectable off-target effects or abnormal liver injury markers [129].

“Modality of delivery” also sets the immunological safety barrier. In canine models of DMD not only transient dystrophin restoration but also sustained expression through AAV mediated Cas9 delivery leads to muscle inflammation and humoral and cytotoxic T-cell responses to Cas9 which are not completely overcome by the use of tissue-specific promoters or short-term steroid immunosuppression, demonstrating that although non-replicating CRISPR systems can be inserted at any locus in a host genome they carry with them their own barriers increasing the complexity of somatic gene editing therapies [130]. In contrast, ECD of CPP-Cas9 RNP complexes enables neuronal editing in the mouse striatum at efficiencies similar to AAV-mediated delivery. whereas it minimizes adaptive immune responses associated with viral vectors. In addition, a high-yield preparation of ultra-low-endotoxin Cas9 further reduces innate immune activation, arguing for transient nonviral RNP delivery as a less immunogenic in vivo CNS editing strategy [131].

The clinical development of therapeutic genome editing is consistently plagued by the inherent trade-off between increasing efficacy and preserving safety margins. Increased editing capabilities can extend the range of targetable mutations, as well increase risks associated with structural variation, immunogenicity and genomic stability over long periods. So technical optimization is not enough to solve all questions. Control approaches towards the safety of cell products are moving away from post-event monitoring and toward front-loaded design, but risk profiles can be very different between cell types and delivery routes, making single solutions hard to find. Finally, whether genome editing can become a routine clinical practice will hinge on the establishment of quantitative and comparable risk–benefit evaluation models that enable the achievement of stable control between therapeutic potential and controllable safety.

### Discussion of safety and ethical issues

As genome editing moves from ex vivo to in vivo, issues of safety are starting to show a kind of multi-dimensional complexity that cannot be summed up into a single dimension of technology. DNA-based gene editing is not only limited to regional target sites but also exhibits a chance of cascading down to the genome architecture, cellular population dynamics, and host immune response levels [132, 133]. As a result, risk assessment needs to move from short-term molecular perturbations to long-term biological effects. Within this framework, safety and ethical considerations are no longer separate, encompassing the controllable boundaries of the risk introduced by technology to broader issues related to clinical decision-making, participant protection and societal acceptance. They deserve both systematic and independent study.

In therapies, the attention of safety checklists moved away from traditional off-target effects toward genomic structural integrity. CRISPR–Cas9-mediated editing through DNA DSBs may also cause nuclear aberrations, including micronuclei and chromatin bridges, which could be associated with target site–associated chromosomal disasters resembling chromothripsis, a catastrophic reshuffling of the genome due to random fragmentation and reassembly [134]. These findings highlight the importance of including chromosomal structural risk factors in standard screening and stratified assessment protocols [122]. Additional in vivo data show that both on-target and off-target sites may yield large (≥ 50‐bp) structural variants, which potentially can be transmitted through the germline to offspring, making SV generation (and its potential heritable risks) an important aspect of both safety review and ethical assessment [135]. For cell-based therapies, modified T cells may carry translocations and large deletions that persist in vivo and expand clonally; there can be selection for viral DNA (engineered virus-like particles [eVLPs]) integration loci during expansion. The results indicate that the long-term genomic stability of reinfused cells and tumorigenic risk control must be in accordance with more strict release criteria, displaying strong and effective long-term monitoring systems [136].

Meanwhile, alongside risk assessment, detection models are developing at breakneck speed. GUIDE-seq facilitates one-pot capture and quantitative profiling of in vivo Cas9 off-targets in mouse liver and lung as well as identification of low-frequency translocations and large deletions, thus providing a quantitative approach for nuclease-based therapy safety testing in vivo [137]. The PE-Associated Chromatin sequencing (PEAC-seq) adds tags at the editing sites and allows for enrichment sequencing to systematically identify off-target events in vitro and in vivo, including DNA translocations with greater genotoxic risk, thus improving detectability of risks typical of structural variants [138]. In the case of PE, PE-tag enables sensitive, genome-wide off-target activity detection in vitro in genomic DNA, mammalian cell lines, and adult mouse liver and uncovers that these activities are largely influenced by pegRNA design-providing a direct handle for safety optimization [139]. Comparative risk analyses have also reported that Cas9 nuclease-induced DSBs can generate large > 100-nt deletions in various human cell lines, where BE and PE are also capable of generating such deletions, but at least 20-fold lower frequency levels that can be further attenuated with the appropriate design principles [140]. BE also exhibits low-frequency, random off-target events broadly distributed across both the genome and transcriptome; consequently, global monitoring must be enhanced and editor specificity continuously maximized for clinical applications [141].

Immunogenicity is another important safety threshold for in vivo editing. Delivery of saCas9 by AAV2 has been reported to produce and present Cas9-derived CD8⁺ T cell epitopes bound to HLA-A 02:01 molecules, resulting in the activation of T cells and killing of transduced cells. This suggests that Cas protein-derived immunotoxicity could limit the therapeutic efficacy, and there is a need for careful choice of vectors, dosing schedules and population stratification [142]. Therapeutic human genome editing is considered ethically permissible on a provisional basis in ethical and theological accounts, but it is considered controversial by the public regarding intergenerational consequences. Furthermore, many issues of human research ethics still remain unresolved such as the problem of adequate informed consent and the autonomy of children, illustrating an immediate need for unambiguous regulatory standards and criteria for review that will minimize risk-spillover on an individual and societal level [143].

Given the transition of therapeutic genome editing toward long-term in vivo applications, safety concerns exhibit substantial structural complexity. Chromosomal translocations, clonal expansion of edited cells, and immune responses against Cas proteins indicate that risks are not confined to immediate molecular outcomes but may evolve over time and potentially exert long-term or even transgenerational effects [144, 145]. This characteristic makes conventional criteria based only or predominantly on off-target rates inadequate to fully describe real-life risks. While high-sensitivity testing has improved the ability to identify risk, it remains unclear what changes are clinically and ethically defensible. Therefore, regulatory flexibility, industrial capacity and commercial potential are three principal limitations that determine the practical application of gene-editing technologies. The maturity of such a platform will have a direct impact on social acceptance as well as environmentally friendly construction of gene-editing tools.

### Regulatory and commercialization barriers

With the development of DNA-based gene-editing technologies from discovery science to product-ready and large-scale applications, challenges related to translational activities have gradually moved away from bench-level research and become more focused at the institutional and industry levels. Given the product modality complexity and the challenge to standardize all aspects of editing, regulators have raised expectations for manufacturing consistency, critical quality attributes (CQA) definition, and long-term safety data for DNA-based gene-editing technologies. Considerable regional variability in the standards and pathways for regulation, combined with difficulties of manufacturing at scale, quality control and cost have dramatically extended timelines to development and uncertainty [146]. In addition, the discrepancy between the rate of clinical information development and that of reimbursement/pricing issues hinders market access efficiency. Therefore, regulatory flexibility, industrial capacity, and commercial potential are three principal limitations that determine the practical application of gene-editing technologies.

CAR-T therapies: Genome-edited T-cell treatments come in parallel with the development of platforms like CAR-T, while this growing product complexity has veered regulatory focus pathways towards manufacturing consistency, CQAs definition safety evaluation frameworks and traceability and operability for control systems [147]. In the context of viral vector routes, starting AAV-based clinical trials in various Asian countries would need to have conformity with varied regulatory and review systems. The development of this template involved a review of the systems used in China, India, Japan, Singapore, Korea, and Taiwan in order to synchronize disparate consultation and review steps which might add to complexity or uncertainty for developers, suggesting the importance for rationalized regulatory chains requiring better coordinated approaches with enhanced regulatory training and engagement at the regional level [148].

The non-clinical evidence chain continues to be the key bottleneck for regulatory approval and general industry progression. Despite accrual overtime of extensive practice in DNA-based gene therapy therapy safety evaluation, there is a paucity of regulatory guidance worldwide. Ongoing uncertainties include species choice, study duration and design and risks of unintended genomic incorporation. There are also emerging safety topics such as re-dosing, paediatric use and reproduction/development toxicity which could in future become nodes of differentiated reviewed risk management for next generation therapies [149, 150]. In the field of immune therapies, Treg therapies are limited by the absence of definitive guidelines and relevant preclinical models [151, 152]. Consequently, investigational new drug (IND) applications of all types depend to a greater or lesser extent on the integrity of the above mentioned pharmacodynamic and biodistribution/safety chains and are less uncertain with mechanisms such as INTERACT and Pre-IND meetings that now exist for early interpretation [153]. On the industrialization side, production process and quality systems are relatively stiff bottlenecks. Cell and DNA-based gene therapy (such as CAR-T cells, viral vectors, or mesenchymal stem cells) suffer from systemic issues with process scaling, maintenance of inter-batch consistency setting of quality control figures and meeting validation paradigms that in turn hinder clinical progression and large scale deployment [154]. Reviews of CAR-T manufacturing also note that worldwide capacity gains are collectively limited by factors such as GMP facilities (facility access, supply chain), automation and process transfer capabilities, QC requirements, and scalability to manufacture the product [155]. Payment and market access pressures compound affordability and accessibility challenges. Scenario simulations of 109 gene therapy trials in advanced-stage development as of 2020 in the US, based on some conservative assumptions, predict annual costs to be around USD 20.4 billion. High priced medicines therefore exert continued pressure on payers’ affordability and patient access, incentivizing the exploration of alternative mechanisms through which to finance such drugs, including instalment payments and outcomes-based reimbursement [156]. Although single-administration gene therapies offer substantial therapeutic potential, uncertainty regarding the durability of long-term benefits—coupled with short-term budgetary shocks—renders rational pricing, management of clinical uncertainty, and budget impact mitigation tightly interlinked challenges. Addressing these issues will require coordinated advances in market instruments and policy reforms to enhance equitable access [157].

The translation of therapeutic DNA-based gene editing in the clinic is at an important tipping point, moving from proof-of-concept to industry production; however, there are several challenges that remain to be addressed. In addition to the technical safety barriers, including DSBs, off-targets and immunogenic responses, there are critical constraints of industrial translation such as impaired scalability of vector manufacturing to a clinical scale, problems with batch-to-batch repeatability in quality control procedures and regulatory diversity that compromise accessibility and commercialization [158]. A comparative scaffold on technical, safety and industrialization dimensions is elucidated in Table 1 that systematically captures the challenges and outcomes associated with genotoxicity, delivery specificity and manufacturing which is supported by corresponding quantitative indicators and their mitigations. This model serves as a decision support tool for optimizing R&D, risk assessment and regulatory communication.

**Table 1:** Key Translational Challenges, Evaluation Metrics, and Mitigation Strategies

Challenge Domain	Specific Issue Description	Potential Consequences/Risk Endpoints	Recommended Evaluation Metrics and Assays	Feasible Mitigation Strategies	Regulatory and Industrialization Implications	References
Off-target and Genotoxicity Risk Control	Structural variants induced by double-strand break (DSB) repair	Unexpected genotoxicity or potential carcinogenic concerns	GUIDE-seq, SITE-Seq, and long-read sequencing	Selection of high-specificity sgRNAs with pre-editing assessment	Requires long-term follow-up and ethical review	[61]
Vector Impurities and Immunogenicity Assessment	Co-packaging of host cell components during vector budding	Uncharacterized immunological effects and toxicity	Systematic characterization of protein and RNA components	Enhanced impurity profiling and pharmacokinetic studies	Indicates the need for additional safety characterization	[159]
In Vivo Targeted Delivery and Specificity	Accompanied by non-specific hepatic uptake	Dose ceiling constrained by hepatotoxicity	Flow cytometric assessment of LT-HSC targeting efficiency	Incorporation of miR-122 target sites to restrict hepatic expression	Further detoxification required prior to translation	[160]
DSB-Associated Targeted Genotoxicity	Cas9 cleavage induces micronuclei and chromatin bridges	Chromosomal fragmentation and rearrangements increase oncogenic risk	Look-Seq single-cell sequencing combined with FISH	Restrict editing to G1 phase and shift toward DSB-free approaches	Requires monitoring of rearrangements and long-term follow-up	[122]
Genotoxicity and Structural Variations	Clustered Regularly Interspaced Short Palindromic Repeats (CRISPR)–Cas9 editing may induce unintended genomic damage, including large deletions and translocations	Genomic instability with increased risk of tumorigenesis or pathogenic mutations	Genotoxicity assays ([Prime Editing-Associated Chromatin sequencing, PEAC-seq], [Chromosomal Aberration Sequence Tagging Sequencing, CAST-Seq]), chromosomal rearrangement and genome integrity analyses	Use of high-fidelity Cas9 variants, optimized gRNA design, DSB-free editors (base/[prime editing, PE]), with long-term surveillance	Genotoxicity concerns represent a key regulatory bottleneck affecting approval and commercialization	[161]
In Vivo Off-target and Genotoxicity Evaluation	In vivo off-target detection is largely indirect and technically complex	Large deletions, translocations, and chromosomal rearrangements	Off-target capture combined with translocation/deletion sequencing	High-fidelity nucleases with optimized guide RNAs	Establishes an in vivo safety evidence chain for release	[137]
CRISPR Off-target and Translocation Identification	Unknown cleavage sites are difficult to label and enrich	Off-targets and translocations induce genotoxicity	PEAC-seq for off-target and translocation detection	PE-based tagging without exogenous dsODN	Strengthens in vivo genotoxicity evidence	[138]
Genome-wide Off-target Specificity Assessment	Current methods rely heavily on indirect predictions	Altered gene expression leading to dysfunction or rearrangements	Insertion-amplification tag sequencing for precise localization	Extended transient delivery of HA to reduce off-targets	Supports regulatory submission of off-target safety	[139]
Cas9-Induced Large Deletion Risk	DSB repair induces deletions of hundreds of base pairs	Elevated genotoxicity limits clinical translation	Long-range amplicon sequencing with ExCas analysis	Use of M4344 or ART558 to suppress deletions	Should be included in release criteria and follow-up plans	[140]
Anti-Polyethylene glycol (PEG) Antibody-Mediated Immune Responses	Anti-PEG antibodies trigger complement activation	Nanocarrier disruption leads to premature release and exposure	Measurement of complement products and C3 deposition	Complement pathway inhibition or antibody-based intervention	Requires anti-PEG screening and immunological QC	[162]
Immunogenicity Associated with lipid nanoparticle (LNP) Delivery	Vaccination-induced elevation of anti-PEG antibodies	Increased systemic reactogenicity and enhanced phagocytosis	Plasma anti-PEG IgG/IgM quantification	Longitudinal monitoring of anti-PEG antibodies and exploration of PEG alternatives	Requires long-term immune surveillance and LNP formulation optimization	[163]
Immunogenicity of PEGylated Liposomes	Administration route influences anti-PEG IgM induction	Repeated administration leads to accelerated blood clearance	Measurement of anti-PEG IgM in mouse serum	Splenectomy partially attenuates IgM production	Include anti-PEG antibody monitoring and route-dependent effects in preclinical evaluation	[164]
Optimization of Off-target Control in Intravenous Delivery	LY6A dependency causes cross-species translational failure	Low central nervous system (CNS) delivery efficiency with increased hepatotoxicity risk	ddPCR-based VCN quantification combined with IHC	Capsid shuffling screening and rational engineering	Neutralizing antibody screening increases translational cost	[165]
Insufficient Mechanistic Understanding of Engineered adeno-associated virus (AAVs)	Limited BBB penetration with unclear mechanisms	High-dose requirements lead to severe adverse events	Human membrane protein microarray screening combined with SPR	Capsid optimization targeting LRP6 while eliminating off-target binding	Mechanistic evidence underpins clinical translation and safety assessment	[166]
Quality Control of mRNA 5′ Cap Structures	Quantification of 5′ caps in long mRNA is challenging	Reduced translation efficiency and enhanced innate immune activation	RNase H cleavage coupled with LC–MS or capillary electrophoresis	TO-based selection, magnetic bead enrichment, and fluorescent labeling	Improves feasibility of release as a critical quality attribute (CQA)	[167]
Scale-up of rAAV Manufacturing Processes	Bubble-induced shear stress in high-density cultures	Cell damage leads to reduced viral titers	Assessment of cell density, viability, LDH, and titer by qPCR	Optimization of agitation and temperature to reduce bubble formation	Ensures production capacity and scalable manufacturing	[168]
Quality Control Testing and Batch Consistency	Lack of standardized quantification of empty/full capsids	Increased dosing volume with safety concerns	Baseline separation and quantification by AEX-HPLC	Development of platform-compatible QC methodologies	Supports product release and in-process control	[169]
Quality Control of rAAV Packaging Characterization	Accurate genome content quantification remains challenging	Elevated empty capsids increase immunogenicity and reduce efficacy	CDMS combined with gel electrophoresis and TapeStation	Thermal incubation reduces heterogeneity and improves purification	Release requires genome-level characterization standards	[170]
AAV Quality Control and Product Release	Accurate determination of empty/full capsid ratios	Aberrant empty capsid ratios compromise efficacy and safety	Size-Exclusion Chromatography coupled with Dynamic Light Scattering (SEC-DLS) dual-wavelength peak area ratio analysis	High-throughput screening using SEC-DLS	Serves as an efficient QC release assay	[171]
Validation and Calibration of AAV Quality Control	Inter-laboratory inconsistency in empty/full capsid quantification	Unidentified impurities increase release risk	SV-AUC discriminates empty, full, and partially filled capsids	Tri-laboratory collaborative calibration of reference standards	Ensures inter-laboratory comparability for QC release	[172]

*Abbreviations:* *AAV* adeno-associated virus, *CAST-Seq*, Chromosomal Aberration Sequence Tagging Sequencing, *CNS* central nervous system, *CQA* critical quality attribute, *CRISPR* Clustered Regularly Interspaced Short Palindromic Repeats, *DSBs* double-strand breaks, *LNP* lipid nanoparticle, *PE* prime editing, *PEAC-seq* Prime Editing-Associated Chromatin sequencing, *PEG* Polyethylene glycol, *SEC-DLS* Size-Exclusion Chromatography coupled with Dynamic Light Scattering

## Future perspectives and innovation

As DNA-based gene editing moves toward clinical maturity, its future trajectory will be shaped not only by technological advances but also by the integration of multidisciplinary innovation and translational feasibility. The following sections outline key dimensions of this evolution, spanning emerging platforms, system-level collaboration, and the shift toward precision medicine.

### Potential and impact of emerging technologies

As DNA-based gene editing advances toward clinical application, innovation is shifting from improving editing efficiency to expanding editable genomic scope, optimizing delivery, and enabling access to previously inaccessible targets. Next-generation systems extend beyond point mutation correction toward programmable genome manipulation and cell-type-specific interventions. These developments substantially broaden the functional and therapeutic landscape of gene editing, particularly for complex diseases. A rigorous understanding of their mechanisms and limitations will therefore be essential for assessing long-term clinical potential and translational feasibility.

The frontier of DNA-based gene editing is shifting from point mutation correction toward programmable gene writing, improved delivery control, and expanded targeting scope. Notably, probabilistic Alignment of Spatial Transcriptomics Experiments (PASTE)-like systems enable site-specific integration of large DNA fragments (up to ~ 50 kb) via a Cas9 nickase–reverse transcriptase–integrase framework, achieving efficient genomic insertion without inducing DNA DSBs or relying on host DNA repair pathways [22, 173, 174]. Researchers have applied this system for insertion of up to 36 kb fragments into human cell lines, primary T cells and non-dividing hepatocytes with high efficiency and few detectable off-target effects, thus extending the known limits of cargo size for Cas9-based and all programmable nuclease-based editing [22]. Naked eVLPs can be used to package and deliver base editors or Cas9 RNPs in vivo. By controlling packaging and release as well as cellular tropism, eVLPs could direct therapeutically relevant editing levels in several tissues with minimal detectable off-target effects, providing proof of concept for a delivery platform that combines the efficiency of viral vectors and the safety of nonviral approaches [159]. PE-eVLPs, loaded with prime editor proteins and gRNAs in a transient RNP form, enable therapeutically relevant precise editing in the retina of mouse models of inherited blindness and partially restoring visual function with minimized off-target effects and decreased risk for transgene integration [175]. Using the RIDE platform, researchers achieved programmable cellular tropism for cell type–specific and temporally defined in vivo delivery of Cas9-RNPs. This strategy rescues disease phenotypes with targeted editing in models of ocular neovascularization and Huntington’s disease, with good tolerability shown in non-human primates [176]. Editor miniaturization has further enhanced vector compatibility: Mini-PE, which employs a compact Cas9 and truncated reverse transcriptase components, reduces the size of the prime editor, enabling AAV–mediated delivery and precise editing in cells and mouse retinas, thereby providing essential engineering support for in vivo PE applications [177].

MtDNA editing is becoming a growth area. DddA-based CBEs (DdCBE) delivered by AAV in vivo achieved site-specific BE of mtDNA in mature tissues such as mouse heart, achieving target editing using both adult and neonatal mice and advancing potential application in the somatic treatment of mitochondrial genetic diseases [178]. HiFi-DdCBE, which achieves targeted modification of the split DddAtox interface, offers enhanced fidelity and efficacy of C → T editing in mtDNA with a reduced background level of bystander mutations, therefore representing a safer and more precise system for rectifying disease-causing mtDNA variants [179]. The TALED system, through optimization at the TadA8e substrate-binding site, markedly reduces transcriptome-wide RNA off-target effects (> 99%), minimizes bystander editing, alleviates cytotoxicity, and prevents embryonic developmental arrest, enabling the generation of mouse models harboring Leigh syndrome–associated pathogenic mtDNA mutations [180]. MitoBEs, which fuse mitochondria-targeted programmable TALE-binding proteins with nickase and deaminase modules, achieve efficient and precise strand-selective A → G or C → T editing of human mtDNA and can correct pathogenic mutations in patient-derived cells [181].

The concerted engineering of editor architecture and delivery for DNA-based gene editing has intertwined boosting target site accessibility, payload writing capability and spatiotemporal eligibility, allowing new technical windows to open into precise intervention of hard-to-reach tissues or complex diseases. These advances not only enhance the accessibility of target sites, but also create a new paradigm for curing complex genetic diseases. To orient technological evolution, Table 2 provides an overview of representative innovative platforms and milestone discoveries that serve to compare their domain advantages and translational difficulties among the various technology strategies. This synthesis provides a scientific basis that should strongly guide choices in technology and risk mitigation for further work. Taken together, in parallel with DNA-level programmable gene writing, sustained innovation in RNA editing and transient modulation strategies is expected to further expand the precision-reversibility spectrum of future therapeutic interventions.

**Table 2:** Milestones of emerging technologies and translational pathways

Innovation Platform/Direction	Representative Technology	Expected Clinical Value & Indications	Key Validation Metrics	Translational Milestones	Major Barriers & Strategic Solutions	References
Engineered pegRNA with stabilized 3′ end	epegRNA incorporating a pseudoknot RNA motif	Enhanced installation and correction efficiency of pathogenic mutations	3–fourfold increase in editing efficiency without increased off-target effects	Validation across multiple cell lines and primary fibroblasts	3′-end degradation leading to low efficiency → structural motif–mediated protection	[35]
Transient in vivo delivery via (prime editing, PE)-(engineered virus-like particles, eVLPs)	PE-eVLP–mediated delivery of PE ribonucleoprotein (RNP) complexes	Genome editing for inherited blindness and (central nervous system, CNS) disorders	Editing efficiency, protein restoration, and ERG outcomes	Therapeutic rescue achieved after a single in vivo injection	Requirement for tissue-targeted envelopes and scalable production	[175]
EXPERT	Extended pegRNA paired with an upstream gRNA	Expanded editing scope for CFTR mutations	Improved efficiency, low indel rates, and whole-genome sequencing–based off-target assessment	Validation across multiple cell types, species, and cystic fibrosis mutations	Instability of long guide RNAs → structural protection and mismatch optimization	[182]
High-throughput prime editing screening platform	PEmax combined with large-scale epegRNA libraries	Functional annotation of small variants to facilitate target discovery	Phenotype-specific epegRNA dropout patterns	Standardized screening of a 240,000-member library	Dependence on mismatch repair (MMR) deficiency and stable editor expression	[183]
Engineering-enhanced pegRNA designs	spegRNA and apegRNA architectures	Improved correction of disease-causing mutations in genetic disorders	On-target editing efficiency and off-target evaluation	Compatibility with PE3/PE5 systems enabling in vivo delivery	Suboptimal efficiency → further pegRNA structural optimization	[184]
Repair pathway modulation to enhance PE	PE4/PE5 systems co-expressing dominant-negative MLH1 (MLH1dn)	Enhanced precision editing for pathogenic mutations	Editing efficiency, indel frequency, and editing purity	Correction of disease-causing mutations in patient-derived induced pluripotent stem cells (iPSCs)	Insufficient MMR suppression → incorporation of MLH1dn	[36]
eVLPs-mediated delivery of PE complexes	v3/v3b PE-eVLP systems	Retinal repair for inherited blindness	Retinal editing efficiency and restoration of visual function	Phenotypic rescue in animal models after a single injection	Limited targeting capacity → envelope engineering	[175]
DNA-free eVLPs	(Base editing, BE) + eVLPs	Gene editing therapies for genetic, ocular, and liver diseases	Editing efficiency, tissue specificity, and off-target editing levels	Efficient, low off-target in vivo editing with vision restoration in retinal disease models	Delivery efficiency, immunogenicity, and long-term efficacy monitoring	[159]
VLP delivery platforms	Virus-like particles	Gene editing therapies and vaccine development	VLP purity, functional delivery, and compositional consistency	Optimization of large-scale manufacturing platforms	Manufacturing consistency and purification efficiency	[185]
Integration of PE with viral systems	Probabilistic Alignment of Spatial Transcriptomics Experiments (PASTE) and PASSIGE systems	Stable integration of full-length therapeutic genes for permanent treatment of monogenic disorders	Gene integration efficiency and long-term expression stability	Preclinical validation of gene integration in animal models	Suboptimal integration efficiency, immune responses, and viral vector–associated risks	[186]

*Abbreviations:* *BE* base editing, *CNS* central nervous system, *eVLPs* engineered virus-like particles, *iPSC* induced pluripotent stem cell, *MLH1dn* co-expressing dominant-negative MLH1, *MMR* mismatch repair, *PASTE* probabilistic Alignment of Spatial Transcriptomics Experiments, *PE* prime editing, *RNP* ribonucleoprotein

### The necessity of interdisciplinary collaboration

Clinical translation of DNA-based gene editing is increasingly recognized as a systems-level challenge rather than a function of single technical advances. Beyond editor efficiency, key determinants include delivery constraints, immunogenicity, biological heterogeneity, and the need for predictive frameworks to ensure reproducibility and safety. Accordingly, gene editing is evolving from molecular tool optimization into an integrated technological ecosystem combining materials science, protein engineering, immunology, multi-omics, and computational modeling. Interdisciplinary integration is therefore critical to enable robust clinical translation and to extend gene editing toward complex diseases and in vivo applications.

As an example of non-viral delivery, LNPs have allowed highly efficient and specific DNA-based BE editing at Pcsk9 in the liver of cynomolgus monkeys, achieving robust reductions on plasma PCSK9 and LDL-C lasting for several months post one single dose administration. This work provides an important proof of concept for “once-and-done” cardiovascular interventions and in vivo editing translation [27]. Protein engineering has produced an iGeoCas9 variant with improved thermostability and activity that, formulated as an RNP-LNP complex, induces both in vivo DNA-based gene editing and HDR in the liver and lung where it reaches 19% editing efficiency of the pathogenic SFTPC gene in lung tissue. The results of the work emphasize the clinical significance of combined “enzyme engineering × nanomaterials × in vivo assay” for the exploitation of therapy indications [187]. Optimization of ionizable lipid facilitated delivery has extended non-viral CRISPR/Cas9 RNP editing efficiency to robust levels in several tumor cell lines and in vivo tumor tissue, establishing a generalizable platform strategy for delivery [6].

With respect to complex organ systems, the designed AAV capsid BI-hTFR1 permits blood–brain barrier transcytosis upon binding human TFR1, resulting in 40–50-fold higher brain expression than with AAV9 in a human TFRC knock-in mouse model and elevated brain/CSF glucocerebrosidase activity after GBA1 delivery—indicating broad CNS penetrance potential [188]. In juvenile rhesus macaques, intratracheal delivery of AAV5 encoding CRISPR/Cas9 resulted in in vivo lung genome editing; pairing this with single-nucleus RNA sequencing identified multicellular transduction lineages and revealed that the correlation of large-animal targeted delivery with cross-scale phenotyping can speed evaluations of clinical feasibility [189]. Safeguarding vectors and promoter design the immune response to AAV-delivered Cas9 has shown age-related differences in the mouse CNS: neonatal delivery results in persistent Cas9 expression but resolving inflammation over time, whereas adult dosing leads to cytotoxic immune clearance with T cell infiltration and antibody production. These data would suggest that pediatric applications need comprehensive engineering of delivery windows, immune modulation and neurotoxicity evaluation [190].

For both delivery modality and safety attributes, eVLPs allow transient delivery of prime editor protein/gRNAs. In 2 genetic models of blinding disease in mice, a single intravitreal treatment resulted in clinically meaningful retinal editing with partial vision restoration without the risks of randomly integrating transgenes [175]. Barcoded gRNA tracking analytics and laboratory-directed evolution resulted in fifth generation eVLPs delivering 2–fourfold enhanced genetic payload to mammalian cells over previous systems, providing a clear demonstration of scalable synergy between vector engineering, screening strategies and process optimization [191]. From an editing paradigm perspective, PASTE modularly combines a Cas9 nickase, reverse transcriptase and serine integrase to achieve targeted integration of large DNA fragments (up to 36 kb) without generating DSB in the DNA or dependence on endogenous repair mechanisms. PASTE displays impressive efficiency and low detectable off-target on different human cells, primary cells, as well as in vivo, shedding new light on for CRISPR safe large-capacity insertions [22]. Similarly, the incorporation of CHANGE-seq with GUIDE-seq for generating large off-target data sets that contain bulged structures, and machine-learning driven generation of models to predict bulge-associated off-targets emphasize a high-throughput experimental approach that is being linked very tightly with computational prediction to allow improved risk-assessment and tool re-design [192].

With the advancement of in vivo gene editing towards translation, a structural tension is manifesting: an iterative improvement of single technological modules that nevertheless must function robustly together within complex environments. Improvement, such as in delivery efficiency or immune response stimulation may need to be balanced against increased off-target effects and safety concerns brought on by broader genome editing; optimizing individual parameters cannot achieve linear improvements in efficacy. This finding implies that gene editing should move beyond a performance-centric tool development paradigm and evolve into an engineering practice where efficiency, safety, and predictability are balanced. Future movement may be more determined not by the constraints of any one technology, but by their ability to form stable and pragmatic reusable system-level synergies across disciplines.

### Prospects for personalized medicine and precision therapy

The era of personalized medicine and precision science has fueled the most precisely targeted clinical applications of DNA-based gene-editing approaches, by facilitating optimal patient-specific interventions based on disease-causing variants, courses of diseases and risk profiles. Unlike conventional population-based therapies, gene editing directly addresses interindividual variability at the molecular level, offering particular advantages in rare genetic disorders and heterogeneous cancers. Recent advances—including in vivo “one-time” editing and personalized cell-based therapies—have entered clinical validation, marking a transition of precision gene editing from conceptual promise to practical implementation.

In a personalized medicine setting, case reports have demonstrated that neonates suffering from a severe form of Carbamoyl Phosphate Synthetase 1 (CPS1) deficiency can be treated in the clinic as a clinical emergency with rapid development and regulatory approval of a patient-specific LNP-delivered DNA-based base-editing therapy. Serious adverse events were not noted after two infusions, but protein tolerance improved and nitrogen scavenger requirements decreased. These data indicate that patient-specific in vivo gene editing is technically possible outside of the laboratory environment, albeit with long-term efficacy and safety still remaining to be evaluated through longitudinal clinical monitoring [63]. Precision strategies targeting disease-causing mutations have also entered clinical trials. EDIT-101 has been evaluated in a phase 1–2 trial of dose escalation in patients with inherited retinal degeneration caused by the CEP290 Intervening Sequence 26 (IVS26) mutation. Use of the treatment in the sub-retinal space was overall safe and some patients showed improvement in cone function, visual acuity, and mobility, which is an argument for further development of genotype-stratified treatments for inherited retinal diseases [64].

Evidence is also increasing for in vivo “one-time” editing for systemic diseases. NTLA-2001, which results in delivery of Cas9 mRNA and a TTR-targeting single-guide RNA encapsulated within LNPs, demonstrated significant dose-dependent decreases in levels of serum TTR following a single administration among patients with hereditary ATTR amyloidosis with polyneuropathy; the majority of adverse events were mild [61]. This target-directed approach has also been applied to ATTR cardiomyopathy: Nex-z reduced serum TTR levels by 89% after a single i.v.-dose at 28 days and by 90% at 12 months. The side-effect profile was predominantly transient infusion reactions and reversible elevations in liver enzymes, and a majority of patients were rated as having stable or improved NYHA functional class [118]. At the population-level intervention/risk management junction, LNP-directed CRISPR base editors have effectively modified PCSK9 in cynomolgus monkeys, achieving near complete suppression of plasma PCSK9 and sustained reduction of LDL-C by around 60% for a period of at least 8 months. These data lend biological rationale to the concept of “once-and-done” precision interventions [27].

Personalized manufacturing strategies with the patient’s own cells also show long-lasting clinical benefits. Two patients with transfusion-dependent β-thalassemia and SCD had high levels of DNA-based editing and pan-cellular increases in fetal hemoglobin extending over a year after receiving their own CD34⁺ hematopoietic stem and progenitor cells following CRISPR targeting of the erythroid specific enhancer region of BCL11A, allowing them to become transfusion-independent and eliminate vaso-occlusive crises [193]. In the solid tumor setting, a phase I trial employed non-viral CRISPR-based precision editing to simultaneously disrupt TRAC/TRBC and knock in patient-specific tumor neoTCRs at the TRAC locus, enabling the generation of individualized T-cell products with successful in vivo trafficking and an acceptable safety profile [75]. In addition, expansion of TILs reactive to tumor neoantigens, combined with knockout of the intracellular immune checkpoint CISH prior to reinfusion, demonstrated feasible safety and signs of potential antitumor activity in metastatic gastrointestinal epithelial tumors, including a sustained complete response in one patient with MSI-H colorectal cancer [194].

Personalized DNA-based editing therapies showcase precision but also generate structural tensions. Patient-customized HTS substantially improves target depth and potential efficacy; however, it also elevates manufacturing complexity, compromises quality control consistency, and raises concerns regarding long-term safety—thus creating an imbalance between clinical benefit and systemic manageability. Despite the advantage of a single dose “in vivo” editing with respect to long-term therapeutic durability, such a class of procedure is associated with significant long term stability and irreversible safety concerns that demand stronger evidence. On the other hand, patient cell–based personalized manufacturing confronts pragmatic trade-offs of controllability with efficacy and access with scalability. Whether customized gene editing will transcend from exceptional scenarios to universally applicable therapeutic archetypes is all going to depend on the collaborative maturation of logged design scaffolds, prompt action metrics, and systematic lifelong follow-up logs. Its development pathway has the capability to redraw both the contours and conceptual underpinnings of precision medicine.

## Discussion

The greater significance of gene editing in disease treatment does not rest in merely enhancing the precision or effectiveness of editing, but rather in reconfiguring medicine’s conceptualization of what constitutes a treatable pathological entity [195]. Traditional therapies primarily modulate downstream signaling pathways or alleviate symptoms, whereas DNA-based editing enables intervention at the level of genetic and transcriptional origins of disease, reframing pathology as aberrant biological information amenable to engineering-based correction. The expanding application of CRISPR systems and derived editing tools across diverse disease models and clinical trials indicates that genome editing has progressed from an experimental concept to a therapeutic modality with a well-defined medical rationale [27, 196]. This transition represents not only a technical advance, but also a paradigmatic shift in therapeutics—one that reshapes how irreversibility, long-term risk, and durability of therapeutic benefit are evaluated. Consequently, the clinical translation of gene editing must be grounded in careful comparisons between short-term efficacy and long-term outcomes and risks.

In this framework, DNA editing and RNA editing constitute two equally important technological directions with distinct yet complementary functional roles. Genome-wide BE and PE enable accurate sequence rewriting with minimal or no reliance on DNA DSBs, making durable correction of monogenic pathogenic mutations an increasingly realistic objective. These approaches have demonstrated near root-cause therapeutic potential in hematopoietic, hepatic, and selected central nervous system disorders, with the principal advantage of achieving permanent phenotypic improvement through one-time molecular reprogramming [117, 197]. By contrast, RNA editing does not aim to permanently alter genomic architecture, but instead enables functional and reversible correction through targeted modulation of transcript sequences, abundance, or splicing patterns. This feature is particularly relevant for neurodegenerative and immune-mediated diseases, where dynamic regulation and reversibility are advantageous. However, inadequate tissue specificity may amplify off-target effects and lead to undesired toxicities, thereby imposing stricter requirements on therapeutic tunability and safety windows; at the same time, the intrinsic reversibility of RNA editing provides an important safety buffer for dose adjustment and temporal control [107, 198–200]. Accordingly, DNA editing and RNA editing should not be viewed as competing technologies, but rather as complementary strategies tailored to different clinical needs—namely, long-term stable repair (through DNA editing) versus adjustable and finely tuned regulation (through RNA editing).

As DNA editing and RNA editing move out of proof-of-concept phase and into realistic clinical applications, the biggest bottleneck lies less with the editing tools themselves and more with structural limitations in delivery capacity and safety thresholds. The actual in vivo delivery efficiency could be the bottleneck determining whether editing can achieve a therapeutically meaningful level, and inadequate tissue specificity may amplify off-target effects and lead to undesired toxicities. More importantly, DSB-based editing approaches have been demonstrated to lead to massive deletion, chromosomal translocation and more complicated genome rearrangements at the target regions. These structural changes can then be expanded and clonally inherited in future generations of cells, with long term biological consequences that are not easily measurable through short- or medium-term proof of mechanism assays [122]. In this light, while the newly developing gene-targeting approaches that do not rely as heavily on DSBs give hope to reducing associated risks in the future, these techniques are still under extensive scrutiny for potential low-frequency genotoxic effects of use in vivo so that long-term safety margins can be explicitly defined [144, 201]. Moreover, immune responses to Cas proteins themselves and viral vectors for delivery limit the safety window for in vivo applications [202]. In conclusion, the risks of gene-editing therapies stem from systemic interactions among editing machineries, delivery modes, and the host biological environment.

DNA editing technologies are not intended to replace existing best practices as a universal solution; rather, they represent a frontier that extends medical intervention into domains where traditional conceptual frameworks are insufficient. Similarly, RNA editing adds flexibility by offering reversible control of gene expression in clinical settings where such dynamic regulation is beneficial. Their ultimate clinical relevance will not be judged by maximal editing efficiency alone, but by whether stable, predictable, and socially acceptable strategies can be implemented in diseases with complex genetic architectures. New editing approaches with high writing capacity and reduced dependence on DNA DSBs, together with reversible RNA-based modulation strategies and low-immunogenicity delivery platforms, collectively expand the therapeutic design space for fine-tuned intervention. Ultimately, the role of gene editing in precision medicine will be determined by the co-evolution of technological maturity, disease biology, and ethical and regulatory systems.

## Data Availability

Not applicable.

## Abbreviations

2pLACE: Two-photon Laser-Assisted Cell Elimination
AAV: Adeno-associated virus
ABCD1: ATP-binding cassette sub-family D member 1
ABE: Adenine Base Editor
ADAR: Adenosine Deaminase Acting on RNA
AHC: Alternating hemiplegia of childhood
ALD: X-linked adrenoleukodystrophy
ALS: Amyotrophic lateral sclerosis
AMBER: Assisted Model Building with Energy Refinement
APOBEC: Apolipoprotein B mRNA Editing Catalytic Polypeptide-like
ApoE: Apolipoprotein E
ASOs: Antisense oligonucleotides
ATTR: Transthyretin Amyloidosis
B2M: β2-Microglobulin
BCL11A: B-cell leukemia/lymphoma 11A
BE: Base editing
CAR-T: Chimeric Antigen Receptor T-cell therapy
CAST-Seq: Chromosomal Aberration Sequence Tagging Sequencing
CBEs: Cytosine base editors
cccDNA: Covalently closed circular DNA
CEP290: Centrosomal Protein 290 kDa
circ-arRNAs: Circular ADAR-recruiting RNAs
CIITA: Class II Transactivator
CISH: Cytokine-Inducible SH2-Containing Protein
CNS: Central nervous system
CPS1: Carbamoyl Phosphate Synthetase 1
CQA: Critical quality attribute
CRISPR: Clustered Regularly Interspaced Short Palindromic Repeats
DdCBE: DddA-derived Cytosine Base Editor
DMD: Duchenne muscular dystrophy
DSBs: Double-strand breaks
eVLPs: Engineered virus-like particles
Gag: Group-specific antigen
GD2: Disialoganglioside 2
GMP: Good manufacturing practice
HAE: Hereditary angioedema
HBBG: Hb G-Makassar
HBBS: Hepatitis B virus binding site
HbF: Fetal hemoglobin
HBG: Hemoglobin Subunit Gamma
HBsAg: Hepatitis B surface antigen
HBV: Hepatitis B virus
HDR: Homology Directed Repair
HITI: Homology-independent targeted integration
HLA: Human Leukocyte Antigen
HSPCs: Hematopoietic stem/progenitor cells
HSV-1: Herpes Simplex Virus type 1
HTT: Huntingtin
ICP: Infected Cell Protein
IND: Investigational New Drug
iPSC: Induced pluripotent stem cell
IVS26: Intervening Sequence 26
KLKB1: Kallikrein B1
LDL: Low-Density Lipoprotein
LDL-C: Low-density lipoprotein cholesterol
LEAPER: Leveraging Endogenous ADAR for Programmable Editing of RNA
LNP: Lipid nanoparticle
LTR: Long Terminal Repeat
MAPT: Microtubule-Associated Protein Tau
Mecp2: Methyl-CpG Binding Protein 2
Mef2c: Myocyte Enhancer Factor 2C
MLH1dn: Co-expressing dominant-negative MLH1
MMR: Mismatch repair
ND: Neurodegenerative diseases
Nex-z: Nexiguran ziclumeran
NRCH: Nuclear localization–regulated Cas9-HF
NTLA: Intellia Therapeutics, Inc.
PAM: Protospacer Adjacent Motif
PASTE: Probabilistic Alignment of Spatial Transcriptomics Experiments
PCSK9: Proprotein Convertase Subtilisin/Kexin Type 9
PE: Prime editing
PEAC-seq: Prime Editing-Associated Chromatin sequencing
PEG: Polyethylene glycol
PHP: Pseudohypoparathyroidism
PVR: Poliovirus Receptor
RESCUE: RNA Editing for Specific C-to-U Exchange
RNP: Ribonucleoprotein
SARS-CoV-2: Severe Acute Respiratory Syndrome Coronavirus 2
SCD: Sickle cell disease
SEC-DLS: Size-Exclusion Chromatography coupled with Dynamic Light Scattering
SNCA: Synuclein Alpha
SOD1: Superoxide Dismutase 1
SREs: Splicing-regulatory elements
TadA8e: Engineered Escherichia coli tRNA adenosine deaminase variant 8e
TALE: Transcription Activator-Like Effector
TALENs: Transcription activator-like effector nucleases
T-ALL: T-cell Acute Lymphoblastic Leukemia
TRAC: T Cell Receptor Alpha Constant region
TRBC: T Cell Receptor Beta Constant region
TTR: Transthyretin
VEGFA: Vascular Endothelial Growth Factor A
ZFNs: Zinc finger nucleases
